# B Cells are Activated via a Nanoporous Interface that Stabilizes Microvilli and Engages Mechanosensitive Ion Channels

**DOI:** 10.1002/advs.76869

**Published:** 2026-08-03

**Authors:** Nozie D. Aghaizu, Willi Weber, Nadine Strempel, Asya Polat, Enrico Klotzsch

**Affiliations:** ^1^ Institute for Biology Experimental Biophysics/Mechanobiology Humboldt Universität Zu Berlin Berlin Germany; ^2^ Experimental and Clinical Research Center (ECRC) at Charité Universitätsmedizin Berlin Berlin Germany; ^3^ Charité Universitätsmedizin Berlin Germany; ^4^ German Center for Neurodegenerative Diseases (DZNE) Berlin Germany; ^5^ Berlin Institute of Health (BIH) at Charité Universitätsmedizin Berlin, BIH Center for Regenerative Therapies (BCRT) Berlin Germany; ^6^ Institute of Active Polymers Helmholtz‐Zentrum Hereon Teltow Germany

**Keywords:** B cell, biology, cell biology, intracellular, ion channel, mechanosensitive channels, nanomaterials, pharmacology

## Abstract

B cell activation typically involves binding of presented antigen by the B cell receptor. Ensuing intracellular signaling with Ca^2+^ mobilization, cytoskeletal remodeling, and transcriptional changes culminates in an appropriate B cell response. Recently, our group developed an antigen receptor‐independent, purely mechanobiological activation platform for T cells. Nanotopological stimulation by exposure to a porous membrane, resulted in robust T cell activation. Whether and how B cells are activated by this substrate on a mechanistic level is presently unknown. Here we report that nanoporous stimulation indeed results in B cell activation, where B cells extended nanopore penetrating microvilli and exhibited activation hallmarks including intracellular Ca^2+^ spikes, phosphorylation of signaling components, and cell surface expression of the activation marker cluster of differentiation (CD) 69, thus recapitulating T cell behavior. This is at least partially mediated by mechanosensitive cation channels, as determined by pharmacology. In summary, our findings demonstrate that B cells are amenable to antigen receptor‐independent activation via nanopores, expanding our knowledge on B cell activation mechanisms.

## Graphical abstract

B cells can be activated independently of antigen recognition via mechanical stimulation through nanoporous substrates. Exposure to such substrates induced B cell microvilli extension into the pores, intracellular Ca^2+^ signaling, phosphorylation of signaling proteins, and CD69 expression. This response was at least partially mediated by Ca^2+^ influx through mechanosensitive cation channels, revealing a mechanobiological pathway for B cell activation.

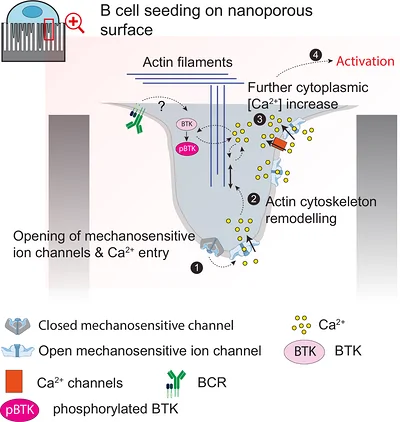

## 1 Introduction

B lymphocytes integrate biochemical and biophysical cues to regulate their activation as well as related B cell responses such as proliferation, isotype switching, differentiation into plasma or memory cells, and cytokine secretion [1, 2, 3, 4]. Conventionally, activation requires engagement of the B cell receptor (BCR), typically following cognate antigen presentation by antigen‐presenting cells (APCs), along with co‐stimulatory input such as CD40 ligation [5, 6]. This results in the initiation of intracellular signaling cascades downstream of the BCR that encompass intracellular Ca^2+^ mobilization, actin cytoskeleton remodeling, and signaling cascade propagation into the nucleus for transcriptional changes via p38, c‐Jun N‐terminal kinase (JNK), extracellular signal‐regulated kinase 1/2 (ERK1/2), nuclear factor kappa‐light‐chain‐enhancer of activated B cells (NFκB), calcium/calmodulin‐dependent protein kinase (CaMK), and nuclear factor of activated T‐cells(NFAT) transcription factors [7, 8, 9]. While these biochemical pathways are well described, accumulating evidence shows that mechanical forces critically shape B cell activation.

APCs are a group of specialized immune cells comprised of dendritic cells (DCs), macrophages, and B cells that is endowed with professional antigen presentation capabilities aimed at for instance, engaging T cells in immune responses. This occurs by presenting to T cells processed antigen peptides on major histocompatibility complex II (MHCII) molecules. However, more generally, B cells can be both at the giving as well as receiving ends of such presentation interactions, the latter of which would be exemplified by follicular DC‐B cell interactions within secondary lymphoid organs [5, 10]. Here, intact antigen is presented to B cells independently of the MHCII antigen processing pathway. B cells interact with antigen presented by APCs via a shared interface called the immune synapse, which is dynamically shaped by actin filament remodeling. Once formed, B cells actively try to enlarge the synapse surface area to maximize productive BCR/antigen interactions by directional flattening toward the synapse [11]. In order to enhance BCR signaling, the synapse subsequently matures by constricting, which locally concentrates ligated BCR molecules. Simultaneously, cortical actin filament is reorganized such that it retains and gathers BCR molecules as well as other signaling components at the synapse by limiting their lateral diffusion. Furthermore, B cells actively generate actin filament‐mediated contractile forces to test antigen affinity in what is called antigen discrimination: high‐affinity interactions between the BCR and antigen presented by APCs withstand pulling by the actin filament‐associated motor protein myosin IIA; this triggers their extraction and internalization into B cells as well as appropriate B cell responses, whereas weaker bonds fail [12, 13, 14, 15]. There are thus extensive mechanobiological contributions to B cell activation relying on cytoskeletal dynamics, traction generation, and membrane deformation [16].

B cell activation further entails intracellular Ca^2+^ signals that encode appropriate B cell responses in accordance with their amplitude and duration [17, 18, 19]. In a two‐step process termed store‐operated Ca^2^^+^ entry (SOCE), Ca^2+^ is first released from the endoplasmic reticulum (ER) following BCR stimulation via PLCγ‐mediated inositol‐1,4,5‐trisphosphate (IP3) generation from the phospholipid phosphatidylinositol 4,5‐bisphosphate (PIP2) and subsequent opening of the Ca^2+^‐conducting IP3 receptor IP3R located on the ER membrane. Depleting ER Ca^2+^ stores are subsequently detected by the ER Ca^2+^ sensors stromal interaction molecule (STIM)1/2, which then translocate to the plasma membrane to trigger a second, more robust increase in intracellular Ca^2+^ by opening Ca^2+^ release‐activated channels (CRAC) made up of ORAI proteins and allowing the influx of Ca^2+^ from the extracellular space. This increase in intracellular Ca^2+^ is integral for intracellular signaling downstream of the BCR and the generation of desired B cell responses.

Evidently, these Ca^2+^ signals also feed back into the mechanobiological framework. Ca^2+^ modulates actin filament dynamic behavior via the Ca^2+^‐dependent kinase calcineurin, which regulates actin rearrangements via cofilin [20, 21, 22]. However, recent reports have implicated Ca^2+^ influx from a different source—Ca^2+^‐conducting mechanosensitive cation channels (MSCs)—in the Ca^2+^/mechanobiology network in B cells. MSCs are a diverse family of ion channels encompassing transient receptor potential vanilloid (TRPV), PIEZO, and hyperosmolality‐gated calcium‐permeable channel / transmembrane protein 63 (OSCA/TMEM63) proteins that convert mechanical stimuli like stretch or touch to cellular responses by allowing the influx of Ca^2+^ from the extracellular space. According to recently published findings, B cell interaction with membrane‐presented antigen increased plasma membrane tension and led to Ca^2+^ influx via MSCs that was distinguishable from typical SOCE‐mediated patterns; both Ca^2+^ influx and activation marker expression could be inhibited by the MSC peptide inhibitor *Grammostola spatulata* mechanotoxin 4 (GsMTx‐4) [23, 24, 25].

How these mechanisms are utilized by lymphocytes exposed to a novel mechanobiology‐based stimulation/activation platform developed by our group (exposure to a nanoporous substrate—an aluminium sheet modified to exhibit pores of 260 nm diameter) is still a matter of active research. This platform was first pioneered for human primary T lymphocytes, which, to some extent, share many of the aforementioned hallmarks of B cell activation [7, 26]. Upon T cell seeding on this substrate and in complete absence of T cell receptor (TCR) stimulation, T lymphocytes exhibited markers of activation including intracellular Ca^2+^ spikes, phosphorylation of signaling cascade proteins, actin cytoskeleton remodeling, and cell surface activation marker expression [27]. These were comparable in extent and magnitude to conventionally used T cell stimulation protocols centered on TCR crosslinking and CD28 co‐stimulation. Mechanistically, these nanoscale topographies stabilized actin filament‐led microvilli protrusions into the nanopores that facilitated TCR clustering, signaling, and SOCE, while simultaneously engaging MSCs, culminating in activation. There are appreciable parallels between T cell/nanopore and T cell/APC interactions, where T cell microvilli protrusions toward and into APC invaginations have been reported to represent hotspots for T cell/APC interactions and enhanced TCR signaling [28, 29, 30].

Whether and to what extent B cells share the capacity to become activated by nanoporous substrate is presently unknown. We thus applied the nanopore stimulation/activation platform to primary human B cells and examined actin‐rich microvilli formation, phosphorylation of proximal signaling proteins (Bruton's tyrosine kinse (BTK), zeta‐chain‐associated protein kinase 70 (ZAP70)), Ca^2+^ dynamics, and CD69 activation marker expression status, together with pharmacological perturbations of BTK signaling, Ca^2+^ homeostasis, actin dynamics, and MSC activity. Our study revealed that B cells do indeed exhibit activation behavior when exposed to a nanoporous substrate, albeit at relatively moderate levels. Mechanistically, this appeared to rely heavily on intracellular Ca^2+^ spikes as well as on actin remodeling to allow microvilli extension into nanopores and phosphorylation of proximal signaling proteins. While these are activation hallmarks known to generally also occur downstream of BCR stimulation, we further identified MSC engagement as a mechanism that exclusively underlay nanopore‐mediated activation, but not that mediated by BCR stimulation. Our study thus extends mechanobiological activation principles from T cells to B cells and could have future implications for B cell/APC modeling and activation‐dependent B cell immunotherapy development.

## 2 Results

### 2.1 B Cells Extend Actin‐Filled Microvilli Into Nanoporous Substrates

To study human primary B cell activatability on nanoporous substrates, we first performed morphological analyses to determine whether B cells exhibit nanopore‐protruding microvilli, which had previously been linked to T cell activatability on nanoporous substrates [27]. To this end, we isolated B cells from human donor‐derived peripheral blood mononuclear cells (PBMCs) by magnetic‐activated cell sorting (MACS) and incubated them on a nanoporous substrate as well as on control substrates for 30 min. A flat aluminium sheet was used as a negative control (henceforth referred to as “flat”) while a flat aluminium sheet coated with antibodies cross‐linking IgM and binding CD40 cell surface molecules on B cells was used as a positive control (“flat (αCD40, αIgM)”).

Exposure to flat surfaces resulted in limited actin filament (F‐actin) structures based on phalloidin labeling, as well as limited phospho‐BTK (pBTK) (B cell activation marker) and immunoglobulin M (IgM) BCR immunolabeling (see representative images in Figure 1A). Conversely, exposure to flat (αCD40, αIgM) surfaces induced filamentous (F‐) actin polymerization coupled with cell spreading, BTK phosphorylation, and the formation of BCR microclusters, which are hallmarks of BCR‐mediated B cell activation (Figure 1B) [9, 31, 32]. Compared with a flat surface, culturing B cells on a nanoporous substrate likewise resulted in increased F‐actin labeling and pBTK immunolabeling even though these signals appeared to be more locally restricted to actin‐rich protrusions into the nanoporous substrate rather than other portions of the cell, akin to what has been observed for T cells [27] (Figure 1C). Similar results were obtained when phosphorylated ZAP70 protein was used as an immunocytochemical B cell activation marker, as was reported elsewhere [33] (Figure S1A–C). By immunocytochemical analysis, we confirmed that these protrusions are of microvillar identity based on the presence of the microvilli marker moesin (Figure S1D,E). While the BCR regulatory cell surface protein CD45 was also expressed on microvilli, its protrusion into nanopores was comparatively smaller compared with that of moesin (phalloidin: 1.2 ± 0.5 µm, moesin: 1.1 ± 0.5 µm, CD45: 0.9 ± 0.4 µm; Figure S1D, E). On average, each B cell exhibited 18.9 ± 2.7 microvilli based on above‐threshold phalloidin labeling (Figure 1D; *N* = 3 biological repeats (independent blood donors), *n* = total 56 cells used for quantification). Typically, these phalloidin^+ve^ protrusions displayed an increased likelihood of harboring pBTK vs. IgM immunolabeling (65.8% ± 14.4% vs. 23.5% ± 6.0%; Figure 1E; unpaired *t‐*test, *p* = 0.0209) despite the fact that IgM was usually present within other parts of the analyzed cells (see representative cell in Figure 1C). This was accompanied by deep pBTK and shallow IgM signal penetration into the nanoporous substrate (phalloidin: 1.2 ± 0.3 µm, pBTK: 0.7 ± 0.1 µm, IgM: 0.3 ± 0.1 µm; Figure 1C,F; one‐way ANOVA, *p* = 0.0066). To exclude the possibility that microvilli formation was an experimental artefact caused by our cell plating protocol that involved low *g*‐force centrifugation to increase the number of substrate‐contacting cells, we compared our standard plating protocol with one that omitted centrifugation during plating and found no significant difference between these two conditions (Figure S1F,G).

**FIGURE 1:**
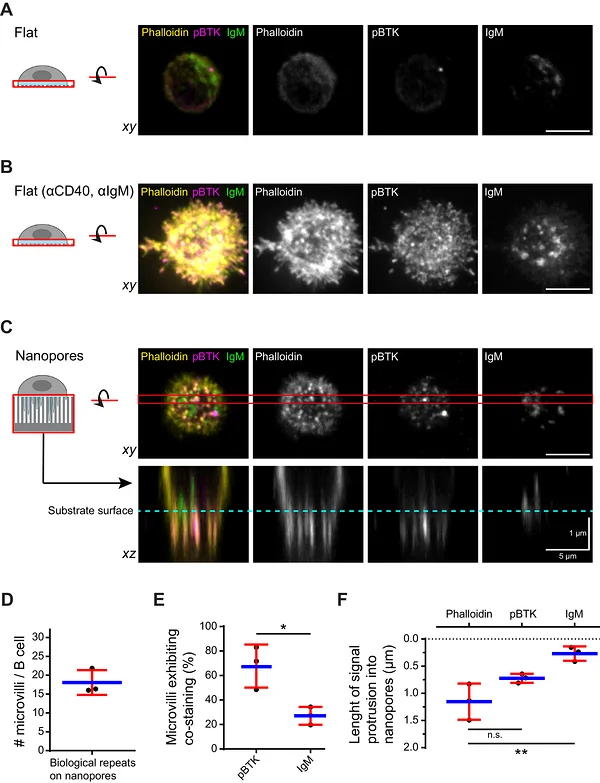
B cells extend actin‐filled microvilli into nanoporous substrates. B cell morphology based on phalloidin staining (yellow) with additional pBTK (magenta) and IgM immunolabeling (green) 30 min after seeding on flat (A), flat (αCD40, αIgM) (B), and nanopore substrates (C). (D) Number of microvilli per B cell. (E) Proportion of phalloidin^+ve^ microvilli co‐stained with activation marker pBTK or the IgM BCR. (F) Average length of phalloidin, pBTK, and IgM signals within phalloidin^+ve^ microvilli. Scale bars, 5 µm (A–C) unless stated otherwise. *N* = 3 biological repeats (independent blood donors), *n* = total 56 cells imaged for microvilli analysis on nanoporous substrate. Data show mean ± SD. Unpaired *t*‐test (E), one‐way ANOVA (F); n.s., not significant; ^*^ *p* < 0.05; ^**^ *p* < 0.01. See also Figure S1.

Together, these data suggest that B cells extend actin‐rich microvilli into nanoporous substrates within 30 min of plating, similar to T cells. While the presence of the activation marker pBTK within such protrusions suggests a degree of B cell activation, the perceived absence of the IgM variant of the BCR indicates a more nuanced involvement of the BCR within the context of microvilli as signaling hubs.

### 2.2 B Cells Exhibit Synchronized Ca^2+^ Responses Upon Contacting Nanoporous Surface

Classically, B cell activation following BCR engagement is known to involve intracellular Ca^2+^ signaling [9, 18, 19, 34, 35]. We hypothesized that intracellular Ca^2+^ may be a general activation conduit in B cells even in BCR‐independent activation paradigms. Thus, to address whether primary human B cells exhibit Ca^2+^ signaling that could be linked to activation upon contacting nanoporous substrates, we performed time‐lapse Ca^2+^ live imaging of B cells exposed to nanoporous substrate vs. flat or flat (αCD40, αIgM) control substrates (*N* = 6 biological repeats; *n* = 1774 cells (flat), 1452 cells (flat (αCD40, αIgM)), 1453 cells (nanopore substrate)).

B cells responded to contact establishment with substrates with three different Ca^2+^ responses irrespective of the type of substrate in use: cells exhibiting no Ca^2+^ spikes, one Ca^2+^ spike, and more than one Ca^2+^ spikes during the time‐lapse live imaging period (representative time‐lapse montages and Ca^2+^ intensity signal traces shown in Figure 2A–F). However, densitometric analysis of B cells grouped according to spiking behavior indicated that, while spiking was observable on flat substrate, this was relatively unsynchronized (Figure 2G). By contrast, spiking behavior of B cells exposed to flat (αCD40, αIgM) substrate was synchronized to be initiated within the first 150 s after cell surface contact (Figure 2H). Crucially, synchronized spiking behavior immediately after contact establishment was recapitulated by B cells on nanoporous substrate, albeit at seemingly decreased stringency compared with flat (αCD40, αIgM) substrate (Figure 2I). This was accompanied by increases in the relative contribution of Ca^2+^ spiking B cells within the overall population following exposure to flat (αCD40, αIgM) as well as nanoporous substrate compared with flat control substrate.

**FIGURE 2:**
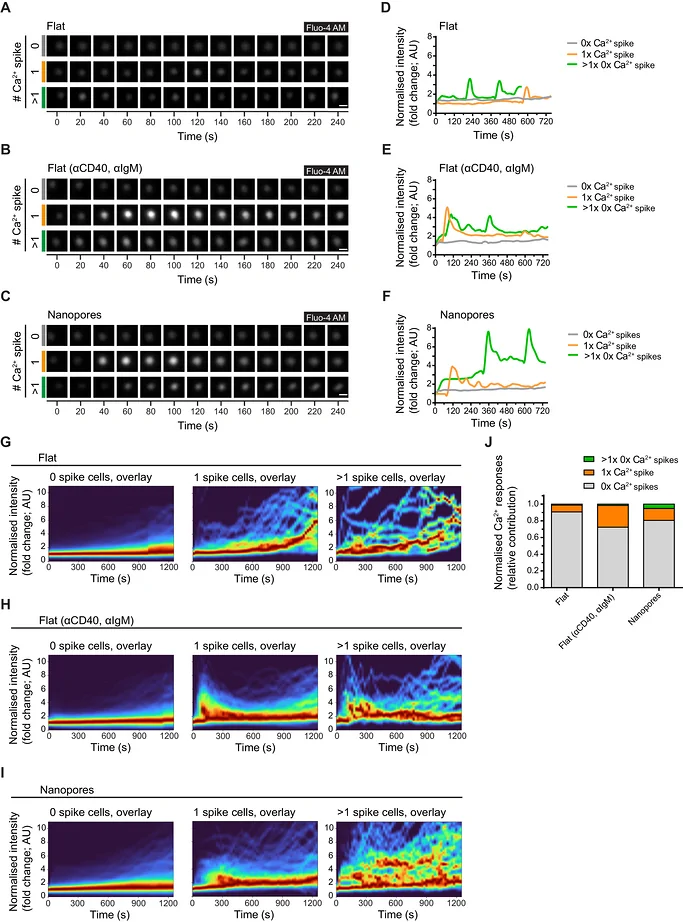
B cells exhibit synchronized Ca^2+^ responses upon contacting a nanoporous surface. Time‐lapse live Ca^2+^ imaging montages of B cells (Fluo‐4 AM, greyscale) upon establishing contact with flat (A), flat (αCD40, αIgM) (B), and nanopore substrates (C). Overlaid example Ca^2+^ signal traces of B cells exhibiting 0 (grey), 1 (orange), or >1 (green) Ca^2+^ peaks upon contact establishment with flat (D), Flat (αCD40, αIgM) (E), and nanopore substrates (F). 2D kernel density estimation of cell population response upon establishing contact with flat (G), flat (αCD40, αIgM) (H), and nanopore substrates (I). (J) Relative contribution of B cells exhibiting 0, 1, or >1 Ca^2+^ spikes upon contact establishment with different substrates. Scale bars, 10 µm (A–C). (G–J) show pooled data from *N* = 6 biological repeats (independent blood donors), *n* = 1774 cells (flat), 1452 cells (Flat (αCD40, αIgM)), 1453 cells (nanopore substrate).

Our data thus demonstrate that B cells indeed display Ca^2+^ signaling behavior following exposure to nanoporous substrate akin to activation‐linked Ca^2+^ signaling triggered by BCR cross‐linking and CD40 ligation. This indicates that nanoporous substrate may have the ability to activate B cells similar to classical activation paradigms.

### 2.3 The Activation Marker CD69 is Upregulated on B Cells After 24 h on Nanoporous Substrate

To further corroborate our finding that B cells are activated by nanoporous substrates as indicated by the Ca^2+^ imaging data (Figure 2), we performed a flow cytometric cell surface expression analysis of B cell activation markers following 24 h or 5 days of incubation on substrates (*N* = 10 and N = 3 biological repeats, respectively; see Figure 3A regarding our used flow cytometry gating strategy).

**FIGURE 3:**
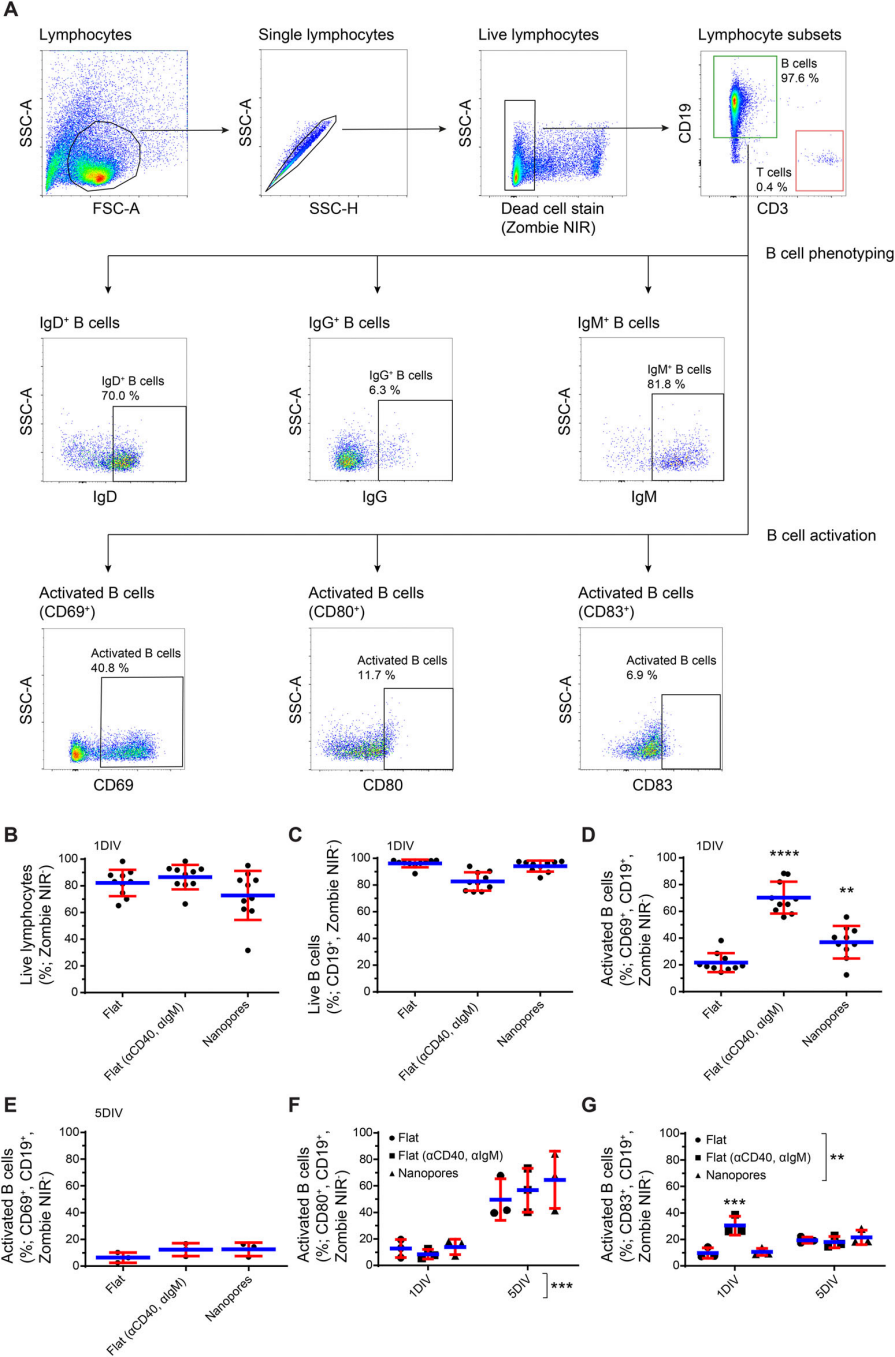
The activation marker CD69 is upregulated on B cells after 24 h on nanoporous substrate. (A) Representative flow cytometry gating strategy for cell surface marker analysis at 24 h on nanoporous substrate. (B) Viability analysis of gated lymphocytes stated as % ZombieNIR^−ve^ of parent single lymphocyte population. (C) Purity assessment of B cells (CD19^+ve^) as opposed to T cells (CD3^+ve^) within cell cultures stated as % of parent live lymphocyte population (see also A). (D) Cell surface expression of the activation marker CD69 on B cells (CD69^+ve^, CD19^+ve^) stated as % of parent live B cell population following 1 day of in vitro incubation. (E) Cell surface expression of the activation marker CD69 on B cells (CD69^+ve^, CD19^+ve^) stated as % of parent live B cell population following 5 days of in vitro incubation. (F) Cell surface expression of the activation marker CD80 on B cells (CD80^+ve^, CD19^+ve^) stated as % of parent live B cell population following one and 5 days of in vitro incubation. (G) Cell surface expression of the activation marker CD83 on B cells (CD83^+ve^, CD19^+ve^) stated as % of parent live B cell population following one and 5 days of in vitro incubation. *N* = 10 biological repeats (independent blood donors) for B–D. *N* = 3 biological repeats for E‐G. One‐way ANOVA (B‐E). Data show mean ± SD. Two‐way ANOVA (F, G); ^**^ *p* < 0.01; ^***^ *p* < 0.001; ^****^ *p* < 0.0001.

Following 24 h of incubation, we observed comparable viabilities irrespective of the substrates that the cells were cultured on, both for gated lymphocytes (flat: 82.2% ± 9.9%; flat (αCD40, αIgM): 86.5% ± 9.1%; nanoporous substrate: 72.8% ± 18.4%; Figure 3B; one‐way ANOVA; n.s.) and for gated CD19^+ve^ B cells (flat: 96.2% ± 2.9%; flat (αCD40, αIgM): 82.7% ± 6.9%; nanoporous substrate: 94.1 ± 4.1%; Figure 3C; one‐way ANOVA; n.s.) with similar values being recorded following 5 days incubation (Figure S2A,B). This suggests that the nanoporous activation paradigm is tolerated by B cells at similarly high levels as conventional activation via BCR cross‐linking and CD40 ligation. In line with the B cell isolation protocol used in this study, the majority of the cultured B cells were IgD and IgM double positive, indicative of a naïve phenotype status (Figure S2C–G). Whereas there appeared to be a significant time point dependent reduction in cell surface IgD levels when comparing B cells cultured for 1 and 5 days in vitro (DIV) (1DIV: flat: 63.6% ± 8.1%, flat (αCD40, αIgM): 73.4% ± 7.8%, nanoporous substrate: 63.6% ± 6.8%; 5DIV: flat: 49.5% ± 3.1%, flat (αCD40, αIgM): 40.0% ± 5.0%, nanoporous substrate: 43.1% ± 3.2%; Figure S2C; two‐way ANOVA, *p*  =  0.4498 (substrates) and *p* = 0.0005 (time points)), cell surface IgM levels were significantly lowered by exposure to activating αIgM antibody on flat (αCD40, αIgM) substrate (1DIV: flat: 72.3% ± 7.4%, flat (αCD40, αIgM): 27.2% ± 17.1%, nanoporous substrate: 68.4% ± 2.8%; 5DIV: flat: 86.7% ± 2.4%, flat (αCD40, αIgM): 6.8% ± 0.8%, nanoporous substrate: 81.0% ± 5.5%; Figure S2E,F; two‐way ANOVA, *p* < 0.0001 (substrates) and *p*  =  0.5359 (time points)). Conversely, B cells predominantly lacked the BCR isotype IgG that is typically associated with a mature phenotype (Figure S2D). We further note that the phenotypic expression of IgD and IgM did not appear to be profoundly altered in activated B cells (compare Figure S2F,G with Figure S2H,I).

Crucially, we detected a significantly increased proportion of B cells expressing the early activation marker CD69 post nanoporous substrate exposure compared with control flat substrate at 1DIV although this increase was modest in comparison to that observed post flat (αCD40, αIgM) substrate exposure (flat: 21.7% ± 7.1%, flat (αCD40, αIgM): 70.3% ± 11.9%, nanoporous substrate: 37.0% ± 12.2%; Figure 3D; one‐way ANOVA; *p* < 0.0001). However, at 5DIV CD69 no longer exhibited any substrate‐dependent expression level changes (flat: 6.3% ± 3.8%, flat (αCD40, αIgM): 12.3% ± 4.8%, nanoporous substrate: 12.6% ± 5.1%; Figure 3E; one‐way ANOVA; *p* = 0.2509). By contrast, the activation marker CD80 was elevated in progressing from 1DIV to 5DIV although there were no substrate‐dependent expression level changes (1DIV: flat: 12.8% ± 6.8%, flat (αCD40, αIgM): 8.5% ± 3.6%, nanoporous substrate: 13.6% ± 5.7%; 5DIV: flat: 49.7% ± 15.6%, flat (αCD40, αIgM): 56.7% ± 16.5%, nanoporous substrate: 64.5% ± 21.6%; Figure 3F; two‐way ANOVA, *p*  =  0.6097 (substrates) and *p*  =  0.0003 (time points)). Conversely, expression levels of the activation marker CD83 were only responsive to flat (αCD40, αIgM) substrate at 1DIV but showed no further dependence on nanoporous substrate or culture duration (1DIV: flat: 9.7% ± 4.0%, flat (αCD40, αIgM): 30.4% ± 7.1%, nanoporous substrate: 10.7% ± 2.7%; 5DIV: flat: 19.5% ± 2.3%, flat (αCD40, αIgM): 18.0% ± 4.3%, nanoporous substrate: 21.6% ± 5.5%; Figure 3G; two‐way ANOVA, *p*  =  0.0031 (substrates) and *p*  =  0.3461 (time points)).

We could thus provide evidence that exposure to nanoporous substrate increases B cell activation both in the short term as well as in a more sustained fashion as demonstrated by Ca^2+^ time‐lapse live imaging and CD69 expression during flow cytometry analysis, respectively, even though this could not be sustained over longer culturing periods. While this corroborates the B cell activation potential of nanoporous substrate, we do note that this potential appears to be inferior to conventional B cell activation via BCR cross‐linking and CD40 ligation. Nonetheless, with CD69 exhibiting expression levels sensitive to nanoporous substrate exposure, this marker was then exclusively used as a B cell activation status readout for the remainder of this study.

### 2.4 Nanoporous Substrates Affect Dendritic Cells Differently Than B Cells

The capacity to become activated by nanoporous substrates appears to be conserved between lymphocytes (B cells and T cells; compare this study with [27]). We next sought to investigate whether this capacity also extended toward all APCs, a group of cells that among other encompasses B cells as well as dendritic cells. To this end, we generated monocyte‐derived dendritic cells (moDCs) cultures from human donor‐derived PBMCs [36, 37] and exposed them to flat, flat (+ve Ctrl) (supplemented with tumor necrosis factor α (TNF‐α), interleukin 1β (IL‐1β), IL‐6 and prostaglandin E2 to cause DC maturation/activation), and nanoporous substrates for 24 h, followed by microscopy and flow cytometry analysis.

We found that, while some moDCs exhibited nanopore‐penetrating protrusions resembling B cell microvilli, the majority of them lacked easily discernible protrusions (Figure S3A–C). We subsequently performed a flow cytometric cell surface expression analysis of moDC activation markers following 24 h incubation on substrates (*N* = 3 biological repeats; see Figure S3D regarding our used flow cytometry gating strategy). We observed comparable viabilities irrespective of the substrates that the cells were cultured on, both for gated leukocytes (flat: 65.2% ± 16.2%; flat (+ve Ctrl): 65.0% ± 14.6%; nanoporous substrate: 59.0% ± 15.3%; Figure S3E; one‐way ANOVA; n.s.) and for gated HLA‐DR^+ve^ moDCs (flat: 97.7% ± 1.2%; flat (+ve Ctrl): 96.3% ± 2.6%; nanoporous substrate: 98.2% ± 0.4%; Figure S3F; one‐way ANOVA; n.s.). This suggests that nanoporous substrate exposure has no overt effect on cell viability.

We next sought to investigate whether nanoporous substrate could stimulate upregulation of known DC maturation/activation markers (CD80, CD83, and CD86). However, we observed no statistically significant differences in cell surface expression levels of CD80 (flat: 44.6% ± 31.4%; flat (+ve Ctrl): 66.2% ± 20.5%; nanoporous substrate: 58.6% ± 21.6%; Figure S3G; one‐way ANOVA; n.s.), while CD83 levels were only significantly increased under positive control but not under nanoporous substrate conditions (flat: 4.1% ± 2.6%; flat (+ve Ctrl): 40.4% ± 2.8%; nanoporous substrate: 13.1% ± 9.7%; Figure S3H; one‐way ANOVA; *p* = 0.0008). Finally, much like CD80, CD86 cell surface expression levels remained unchanged under our experimental conditions (flat: 75.1% ± 10.3%; flat (+ve Ctrl): 85.8% ± 9.1%; nanoporous substrate: 74.1% ± 11.1%; Figure S3I; one‐way ANOVA; n.s.).

While our data do not unequivocally exclude the potential of moDCs to enter a maturation/activation phenotype upon nanoporous substrate exposure, this could presently not be achieved using the current experimental conditions. This indicates that activatability does not appear to be a characteristic that is fully conserved between APCs.

### 2.5 B Cell Microvilli Protrusion Into Nanopores Requires Intracellular Ca^2+^ and Dynamic Actin Cytoskeleton Remodeling

We next sought to determine the mechanisms underlying nanopore‐mediated B cell activation and whether this is distinct from classically described BCR‐mediated B cell activation. To this end, we used a compact, data‐inspired as well as literature‐anchored pharmacological screen to shed light on the role of pre‐selected potential signaling components in nanopore‐mediated B cell activation (Figure 4). We initially focused on the role of BTK [38, 39], intracellular Ca^2+^ [19, 34, 35], and actin cytoskeleton dynamic behavior [20, 31, 40, 41, 42, 43]. BTK is a well‐described signaling hub downstream of the BCR, while we speculate that respectively increasing or decreasing intracellular Ca^2+^ and actin cytoskeleton rearrangements may promote B cell activation via microvilli or block activation by attenuating microvilli.

**FIGURE 4:**
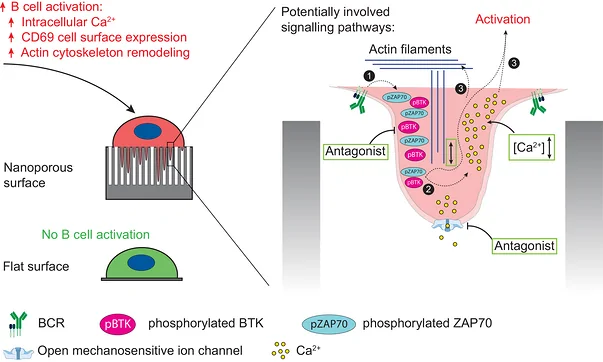
Schematic illustrating rationale behind experimental design of pharmacological screening. Typically, BCR‐mediated B cell activation entails intracellular cell signaling pathways involving, among other aspects, phosphorylation of, and further signaling propagation by, BTK and ZAP70 (1), Ca^2+^ signaling (2), and actin cytoskeleton remodeling (3). These signaling components were chosen as targets in our pharmacological screening to determine their potential role in nanopore‐mediated B cell activation (see green text boxes). In addition, mechanosensitive cation channels were also targeted.

We first performed a morphological assessment of B cells following a 1‐h pre‐incubation period in the presence of pharmacological agents and subsequent incubation on a nanoporous substrate for 30 min (see Figure 5 figure legend for description of *N*, *n*). Compared with the dimethyl sulfoxide (DMSO) vehicle control, the BTK‐selective inhibitor Ibrutinib [44, 45] had no significant effect on the number of microvilli per cell (DMSO: 18.9 ± 2.7 vs. Ibrutinib: 14.0 ± 9.2; Figure 5A–C; unpaired *t*‐test, n.s.) or the length of microvilli based on phalloidin, pBTK, and BCR (IgM) signal protrusions into the nanoporous substrate. Under DMSO conditions, phalloidin, pBTK, and IgM signals extended 1.2 ± 0.3 µm, 0.7 ± 0.1 µm, and 0.3 ± 0.1 into the nanopores, respectively. Ibrutinib treatment did not significantly alter these distributions (phalloidin: 1.2 ± 0.2 µm, pBTK: 0.9 ± 0.0 µm, IgM: 0.4 ± 0.1 µm; Figure 5D; one‐way ANOVA, n.s.). Likewise, pBTK immunolabeling signal intensities remained unchanged (DMSO: 57.9 ± 32.2 vs. Ibrutinib: 71.8 ± 29.2; Figure 5E; unpaired *t*‐test, n.s.).

**FIGURE 5:**
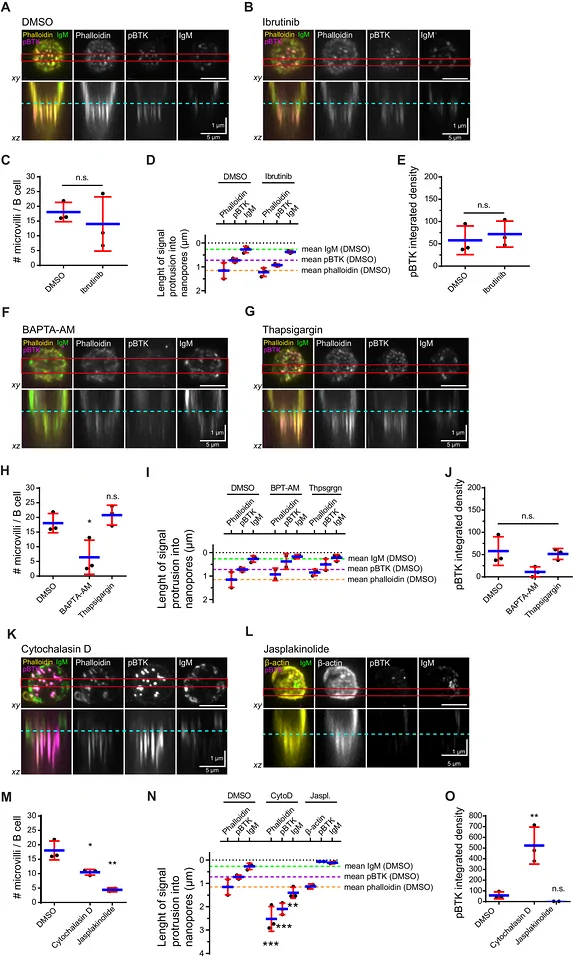
B cell microvilli protrusion into nanopores requires intracellular Ca^2+^ and dynamic actin cytoskeleton remodeling. B cell morphology based on phalloidin staining (yellow) with additional pBTK (magenta) and IgM immunolabeling (green) following incubation with pharmacological agents for 1 h and subsequent plating on substrates for 30 min prior to fixation, IHC, and microscopy. Representative B cells treated with DMSO (A) and Ibrutinib (B), as well as respective quantifications of microvilli numbers per cell (C), average length of phalloidin, pBTK, and IgM signals within phalloidin^+ve^ microvilli (D), and pBTK intensity quantification within phalloidin^+ve^ microvilli (E) are shown. Representative cells treated with BAPTA‐AM (F) and Thapsigargin (G) as well as respective quantifications of microvilli numbers per cell (H), average length of phalloidin, pBTK, and IgM signals within phalloidin^+ve^ microvilli (I), and pBTK intensity quantification within microvilli (J) are shown. Representative cells treated with Cytochalasin D (K) and Jasplakinolide (L) as well as respective quantifications of microvilli numbers per cell (M), average length of phalloidin, pBTK, and IgM signals within phalloidin^+ve^ microvilli (N), and pBTK intensity quantification within phalloidin^+ve^ microvilli (O) are shown. Bottom panels in (A, B, E, F, I, J) show a sideways view (*xz*) of boxed ROIs in upper panels (*xy*), with a turquoise dotted line demarcating the surface of nanoporous substrates. Scale bars, 5 µm (A, B, F, G, K, L) unless stated otherwise. *N* = 3 biological repeats (independent blood donors), *n* = total 56 cells (DMSO), 58 (Ibrutinib), 56 (BAPTA‐AM), 64 (Thapsigargin), 48 (Cytochalasin D), 25 (Jasplakinolide), 57 (GsMTx‐4), 55 (GsMTx‐4, Thapsigargin). Data show mean ± SD. Unpaired *t*‐test (C, E), one‐way ANOVA (D, H‐ J, M‐O); n.s., not significant; ^*^ *p* < 0.05; ^**^ *p* < 0.01; ^***^ *p* < 0.001.

By contrast, manipulating intracellular Ca^2+^ levels did impact B cell microvilli. While increasing the intracellular Ca^2+^ concentration via Thapsigargin‐mediated Ca^2+^ re‐uptake inhibition into intracellular stores had no effect on the number of microvilli, chelation of intracellular Ca^2+^ with 1,2‐bis(o‐aminophenoxy)ethane‐N,N,N',N'‐tetraacetic acid tetra(acetoxymethyl) ester (BAPTA‐AM) resulted in a significant reduction (DMSO: 18.9 ± 2.7, BAPTA‐AM: 6.4 ± 5.8, Thapsigargin: 20.8 ± 3.4; Figure 5F–H; one‐way ANOVA, *p* = 0.0143). However, the length of microvilli remained unchanged. Under control (DMSO) conditions, phalloidin, pBTK, and IgM respectively protruded 1.2 ± 0.3 µm, 0.7 ± 0.1 µm, and 0.3 ± 0.1 µm into the nanopores. Neither BAPTA‐AM (phalloidin: 0.9 ± 0.3 µm, pBTK: 0.4 ± 0.3 µm, IgM: 0.2 ± 0.1 µm) nor Thapsigargin (phalloidin: 0.8 ± 0.1 µm, pBTK: 0.5 ± 0.2 µm, IgM: 0.2 ± 0.1 µm) significantly affected these distributions (Figure 5H; one‐way ANOVA, n.s.). However, we did observe respective reductions and increases in immunolabeling intensity of the activation marker pBTK following BAPTA‐AM and Thapsigargin treatment (see representative images in Figure 5F,G) even though these changes were not statistically significant (DMSO: 57.9 ± 32.2, BAPTA‐AM: 11.1 ± 11.5, Thapsigargin: 51.4 ± 12.3; Figure 5J; one‐way ANOVA, n.s.).

We then looked into the role of dynamic actin cytoskeleton rearrangements by applying drugs that have been described to either block actin filament polymerization, thus typically resulting in net filament destabilization (Cytochalasin D), or promote polymerization and hence actin filament structures (Jasplakinolide). We note that Jasplakinolide and phalloidin compete for the same binding site on monomeric actin, due to which phalloidin staining was replaced by immunolabeling for β‐actin. We found that Cytochalasin D and Jasplakinolide significantly reduced the number of microvilli per B cell (DMSO: 18.9 ± 2.7, Cytochalasin D: 10.5 ± 1.0, Jasplakinolide: 4.4 ± 0.7; Figure 5K–M; one‐way ANOVA, *p* = 0.0068). It was also readily apparent that, despite their reduced numbers, those microvilli on B cells exposed to Cytochalasin D were significantly longer than those of cells exposed to DMSO control (Figure 5N); by contrast, those few remaining microvilli structures in cells treated with Jasplakinolide were merely non‐significantly shortened (Figure 5N), while most of the β‐actin immunolabeling appeared lamellipodial in nature (Figure 5L). Under DMSO conditions, phalloidin, pBTK, and IgM signals respectively extended 1.2 ± 0.3 µm, 0.7 ± 0.1 µm, and 0.3 ± 0.1 µm into the nanopores. Cytochalasin D treatment increased these distances to 2.5 ± 0.5 µm, 2.1 ± 0.2 µm, and 1.4 ± 0.2 µm, whereas Jasplakinolide treatment reduced them to 1.1 ± 0.1 µm (β‐actin), 0.1 ± 0.0 µm (pBTK), and 0.1 ± 0.0 µm (IgM) (Figure 5N; one‐way ANOVA, *p* < 0.0001). Interestingly, we observed a striking increase in immunolabeling intensity of the activation marker pBTK in Cytochalasin D‐treated cells and a reduction in Jasplakinolide‐treated cells (DMSO: 57.9 ± 32.2, Cytochalasin D: 523.5 ± 172.9, Jasplakinolide: 2.1 ± 0.1; Figure 5O; one‐way ANOVA, *p* = 0.0051). We note that Cytochalasin D and Jasplakinolide treatment of B cells has been linked in the published literature to increases in F‐actin and intracellular Ca^2+^, which itself is linked to activation, even in the absence of BCR engagement [17, 46, 47, 48].

We thus find that if there is indeed a mechanistic link between either the formation of microvilli or signaling through microvilli and B cell activation on nanoporous substrates, this may be at least partially guided by intracellular Ca^2+^ signaling as suggested by manipulations of intracellular Ca^2+^ levels and their consequences on microvilli numbers and pBTK immunolabeling. Somewhat counterintuitively, Cytochalasin D, while reducing microvilli numbers, markedly increased levels of the activation marker pBTK, whereas Jasplakinolide reduced both microvilli numbers and pBTK levels. These results call for further dissection of the role of intracellular Ca^2+^ and actin filament rearrangements on nanopore‐mediated B cell activation.

### 2.6 Nanopore‐Mediated B Cell Activation Involves Cytoplasmic Ca^2+^ and Actin Dynamics

To further investigate how signaling through BTK, intracellular Ca^2+^, and actin cytoskeleton dynamic behavior affect B cell activation, we performed a flow cytometric cell surface expression analysis of the B cell activation marker CD69 following 24 h incubation on nanoporous and control substrates (*N* = 3 biological repeats). While some activation mechanisms may well be conserved between BCR‐ and nanopore‐mediated activation, we were specifically interested in mechanisms that exclusively affect nanopore‐mediated B cell activation.

With the exception of the intracellular Ca^2+^ chelator BAPTA‐AM, all pharmacological agents were generally well tolerated by gated lymphocytes or gated CD19^+ve^ B cells on flat, flat (αCD40, αIgM) and nanoporous substrates according to viability assessments (Figure S4). This likely reflects the importance of intracellular Ca^2+^ in various B cell processes, including cell survival. Compared with DMSO, blocking signaling through BTK with Ibrutinib significantly decreased the number of CD69^+ve^ B cells on flat (αCD40, αIgM), while this reduction was not significant on nanoporous substrate (Figure 6A; one‐way ANOVA, *p* < 0.0001).

**FIGURE 6:**
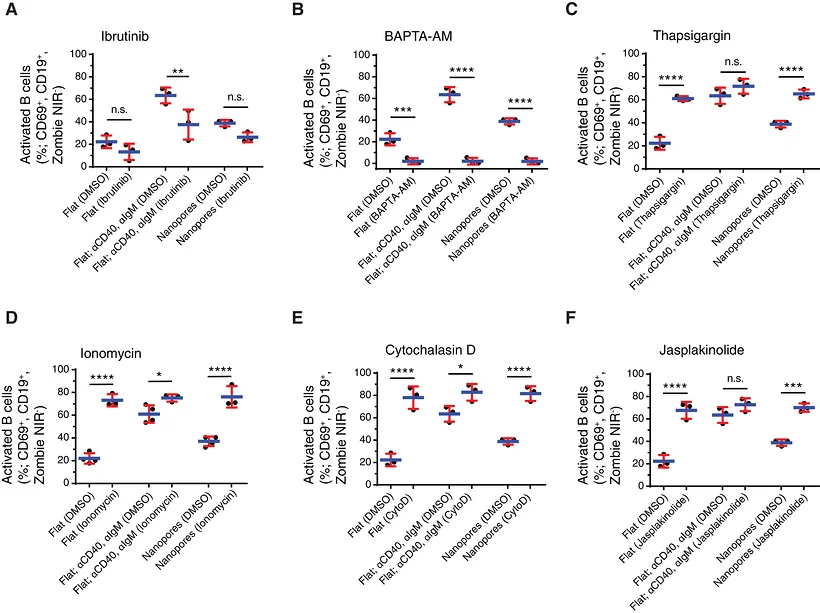
B cell activation on nanopores is *not* exclusively mediated by pBTK, general cytoplasmic Ca^2+^ signaling, or actin cytoskeleton dynamic behavior. Cell surface expression of the activation marker CD69 on B cells (CD69^+ve^, CD19^+ve^) stated as % of parent live B cell population following incubation on flat, flat (αCD40, αIgM), and nanopore substrates in presence of Ibrutinib (A), BAPTA‐AM (B), Thapsigargin (C), Ionomycin (D), Cytochalasin D (E), and Jasplakinolide (F). *N* = 3 biological repeats (independent blood donors). Data show mean ± SD. One‐way ANOVA (A–E); n.s., not significant; ^*^ *p* < 0.05; ^**^ *p* < 0.01; ^***^ *p* < 0.001; ^****^ *p* < 0.0001. See also Figures S4A–P and S5A,B.

Intracellular Ca^2+^ sequestration using BAPTA‐AM led to a near‐complete elimination of the CD69^+ve^ B cell population on flat, flat (αCD40, αIgM), and nanoporous substrates compared with the DMSO control (Figure 6B; one‐way ANOVA, *p* < 0.0001). By contrast, increasing intracellular Ca^2+^ levels with Thapsigargin did not further increase the number of CD69^+ve^ B cells on the positive control flat (αCD40, αIgM) substrate indicative of an activation ceiling under these conditions. Thapsigargin did however, significantly increase the proportion of CD69^+ve^ B cells on nanoporous and flat substrates to levels observed on flat (αCD40, αIgM) (Figure 6C; one‐way ANOVA, *p* < 0.0001). We further corroborated these findings by using the Ca^2+^ ionophore ionomycin, which similarly elevates cytosolic Ca^2+^ levels, albeit with a different mechanism of action (Figure S5). Further increases in the fraction of CD69^+ve^ B cells were observed across all substrate conditions compared with the DMSO control (Figure 6D; one‐way ANOVA, *p* < 0.0001). Together, these data suggest that Ca^2+^ is necessary and sufficient for B cell activation, which fits our imaging findings in Figure 5E–H.

We then investigated how manipulations of actin filament dynamic behavior with Cytochalasin D and Jasplakinolide impact CD69 expression on B cells. In keeping with our imaging data where we detected markedly increased pBTK activation marker signal (Figure 5I), Cytochalasin D significantly expanded the population of CD69^+ve^ B cells on flat, flat (αCD40, αIgM), and nanoporous substrates compared with DMSO control (Figure 6E; one‐way ANOVA, *p* < 0.0001). Surprisingly, Jasplakinolide treatment similarly expanded the population of CD69^+ve^ B cells on flat and nanoporous substrates compared with DMSO control, while the increase was not significant on flat (αCD40, αIgM) (Figure 6F; one‐way ANOVA, *p* < 0.0001). This is in spite of the fact that Jasplakinolide markedly decreased pBTK activation marker signal and microvilli numbers in our imaging data (Figure 5J).

Taken together, our flow cytometry data point toward a limited involvement of BTK while Ca^2+^ and actin cytoskeleton rearrangement take on a central role in nanopore‐mediated B cell activation, even though the exact mechanism of the latter is yet to be determined. However, these involvements appear to be generally conserved components of activation as they impacted B cells on nanoporous substrates as well as control substrates alike.

### 2.7 Mechanosensitive Cation Channels Exclusively Mediate Nanopore‐Mediated B Cell Activation but not Activation via CD40 and IgM Crosslinking

In a continued search for a mechanistic description of nanopore‐specific B cell activation, our attention then shifted to mechanosensitive cation channels (MSCs), which, as a collective of channels, are known for their ability to sense cellular forces [49]. We reasoned that the extension of microvilli into nanopores introduced a change in the degree of membrane tension conducive to MSC opening, Ca^2+^ influx through these channels, and subsequent B cell activation. To test this, we treated B cells with GsMTx‐4, a peptide toxin that inhibits MSCs [24], for 1 h followed by incubation on substrates for 30 min for microscopic analyses (*N* = 3 biological repeats, *n* = 57 cells) or 24 h for flow cytometric analysis (*N* = 3 biological repeats).

We determined that blocking MSCs with GsMTx‐4, and thus attenuating their Ca^2+^ conductance, significantly reduced the number of microvilli extended by B cells into nanoporous substrate; this metric was not restored even in additional presence of Thapsigargin to elevate intracellular Ca^2+^ levels (DMSO: 18.9 ± 2.7, GsMTx‐4: 7.9 ± 1.3, GsMTx‐4/Thapsigargin: 10.5 ± 2.3; Figure 5A and 7A–C; one‐way ANOVA, *p* = 0.0053). Microvilli length based on phalloidin, pBTK, and IgM signal protrusions into nanopores remained unchanged. Under control (DMSO) conditions, phalloidin, pBTK, and IgM respectively protruded 1.2 ± 0.3 µm, 0.7 ± 0.1 µm, and 0.3 ± 0.1 µm into the nanoporous substrate. Treatment with GsMTx4 (phalloidin: 0.8 ± 0.1 µm, pBTK: 0.7 ± 0.2 µm, IgM: 0.2 ± 0.0 µm) or with GsMTx4/Thapsigargin (phalloidin: 0.7 ± 0.1 µm, pBTK: 0.5 ± 0.1 µm, IgM: 0.3 ± 0.1 µm) did not significantly alter these distributions (Figure 7D; one‐way ANOVA, n.s.). We also did not observe any significant changes in pBTK immunolabeling intensity following GsMTx‐4 or GsMTx‐4/Thapsigargin treatment (DMSO: 57.9 ± 32.2, GsMTx‐4: 74.0 ± 50.2, GsMTx‐4/Thapsigargin: 81.2 ± 84.0; Figure 7E; one‐way ANOVA, n.s.).

**FIGURE 7:**
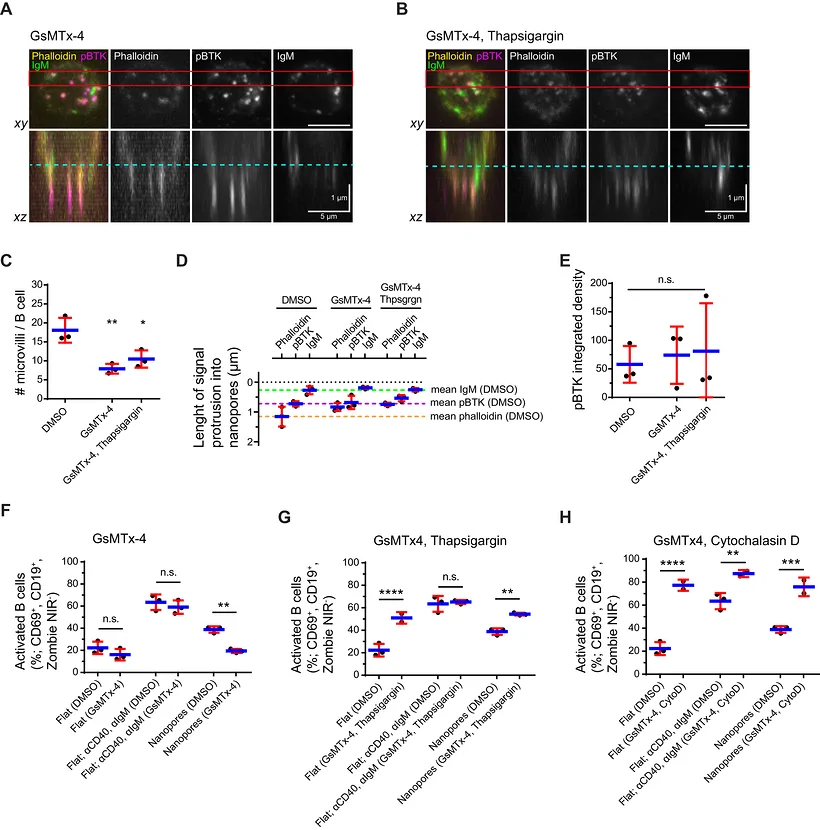
Mechanosensitive cation channels exclusively mediate nanopore‐mediated B cell activation but not activation via CD40 and IgM crosslinking. B cell morphology based on phalloidin staining (yellow) with additional pBTK (magenta) and IgM immunolabeling (green) following incubation with GsMTx‐4 (A) and GsMTx‐4/Thapsigargin (B) for 1 hr and subsequent plating on substrates for 30 min prior to fixation, IHC, and microscopy. Bottom panels in (A, B) show a sideways view (*xz*) of boxed ROIs in upper panels (*xy*), with a turquoise dotted line demarcating the surface of nanoporous substrates. (C) Microvilli numbers per cell. (D) Average length of phalloidin, pBTK, and IgM signals within phalloidin^+ve^ microvilli. (E) pBTK intensity quantification within phalloidin^+ve^ microvilli. Cell surface expression of the activation marker CD69 on B cells (CD69^+ve^, CD19^+ve^) stated as % of parent live B cell population following 24 h incubation on flat, flat (αCD40, αIgM), and nanopore substrates in the presence of GsMTx‐4 (F), GsMTx‐4/Thapsigargin (G), GsMTx‐4/Cytochalasin D (H). Scale bars, 5 µm (A, B) unless stated otherwise. *N* = 3 biological repeats (independent blood donors), *n* = total 57 cells (GsMTx4), 55 (GsMTx‐4/Thapsigargin). Data show mean ± SD. One‐way ANOVA (C‐H); n.s., not significant; ^*^ *p* < 0.05; ^**^ *p* < 0.01; ^***^ *p* < 0.001.

We then sought to investigate whether the reduction in microvilli numbers brought about by GsMTx‐4 translated into a reduction in activation marker expression as determined by flow cytometry. Indeed, MSC inhibition by GsMTx‐4 resulted in the significant reduction of the CD69^+ve^ B cell population specifically on nanopores, while this had no effect on CD69^+ve^ B cells on flat and flat (αCD40, αIgM) substrates (Figure 7F; one‐way ANOVA, *p* < 0.0001; see also viability readouts in Figure S4F–H,N–P). This finding thus demonstrates that MSCs are important specifically for nanopore‐mediated B cell activation and not activation mediated by BCR crosslinking and CD40 ligation. Since MSC function is intrinsically linked to their Ca^2+^ conductance, we performed control experiments to re‐supply intracellular Ca^2+^ in B cells treated with GsMTx‐4 from non‐MSC sources. Indeed, the presence of Thapsigargin or Cytochalasin D in addition to that of GsMTx‐4 restored the CD69^+ve^ B cell population on nanoporous substrates (compare “Nanopores” treatment groups in Figure 7F–H).

Taken together, these novel findings demonstrate that nanopore‐mediated B cell activation engages MSCs and Ca^2+^ influx through these channels. This activation mechanism appears to be distinct from activation via BCR crosslinking and CD40 ligation, where such MSC engagement is not supported by our data.

## 3 Discussion

Classically, B cell activation has been thought to require BCR detection of antigens typically presented to them via APCs such as follicular DCs, an interaction that occurs in secondary lymphoid organs. This triggers a cytoplasmic signaling cascade that includes the intracellular messenger Ca^2+^, cytoskeletal remodeling especially around the B cell/APC interface, and changes in gene expression related to the exact nature of the B cell stimulus and the associated B cell response [2]. This present study demonstrates the ability of primary human B cells to become activated in a manner that relies on mechanobiological phenomena in the complete absence of antigen or APCs. The mechanobiological stimulus consisted of exposure to a nanoporous substrate [27]. In our experimental setup, B cells exhibited markers of activation following contact establishment with this substrate, including intracellular Ca^2+^ spikes, phosphorylation of intracellular signaling components associated with activation (BTK, ZAP70), and upregulation of the cell surface molecule CD69.

Nanopore‐mediated activatability is thus a property that B cells share with T cells. T cell activation on a nanoporous substrate was previously reported by our group [27] and can be embedded within a wider framework of the growing field of T cell mechanobiology that investigates T cell force sensation, potentiation, and translation with respect to interactions with other cells or tissues, the extracellular space, and the blood circulation [50, 51]. T cells react robustly to nanopore stimulation, resulting in intracellular Ca^2+^ responses and CD69 expression levels that matched the high levels of a conventional stimulation paradigm via TCR cross‐linking and CD28 ligation. While our group previously posited that CD45 is excluded from nanopore‐penetrating microvilli in our original report [27], an updated experimental protocol, including improved characterization of the nanoporous substrate challenged this view as CD45 was readily observable in T cell microvilli [52]. By contrast, we observed only modest B cell activation on nanoporous substrate compared with a conventional B cell activation stimulus consisting of BCR cross‐linking and CD40 ligation. Whether this comparatively modest activatability reflects limitations intrinsic to B cells or the need for protocol optimizations remains to be determined. However, we note that the same nanopore stimulation protocol was used in this present B cell study that had previously been developed and optimized by our group for T cells [27, 52] and that an optimum nanopore diameter of 260 nm for B cell activation had previously been experimentally determined (Figure S6A,B). Much like T cells, B cells exhibited CD45 within nanopore‐penetrating microvilli (Figure S1D), although it is still a matter of ongoing research how this affects their activation at the cell signaling level. The perceived incomplete penetration of CD45 down the entire length of microvilli (Figure S1D,E) may locally segregate the inhibiting phosphatase activity of CD45 from inherent tonic BCR signaling at the tip of microvilli, analogous to CD45 tip exclusion from T cell microvilli that can locally amplify TCR signaling [53]. In light of the seemingly restricted access of the IgM BCR isotype to B cell microvilli (Figure 1C,E,F), future studies should address whether the equally expressed but much smaller IgD isotype achieves better microvilli penetration and thus BCR signaling at CD45‐lacking microvilli tips. Nonetheless, there thus appears to be a level of mechanistic conservation pertaining to nanopore substrate‐mediated activation within the lymphocyte lineage. However, while B cells also belong to the group of APCs that further encompasses DCs and macrophages, we failed to detect nanopore‐activatability in moDCs. For that reason, lymphocyte activation on nanopores is unlikely to be a generalized, stress‐dependent activation response, although this view could be challenged in the future with moDC‐favorable modifications in the experimental protocol such as nanopore diameter and length of nanopore substrate exposure.

In this present study, we examined B cell activation on a nanoporous substrate using a panel of experimental readouts at three different time points: Ca^2+^ time‐lapse live imaging, morphological assessment by fluorescence microscopy, and CD69 expression by flow cytometry at the time of contact establishment, 30 min after plating, or 1‐5 days after plating on substrate. Future studies should aim to expand the characterization of B cell activation behavior on nanopores. Whether B cells can be activated by nanoporous substrate beyond 24 h is presently unclear and should be addressed given that many B cell responses to external stimuli can operate on longer time scales. The list of responses includes proliferation, isotype switching, differentiation into plasma or memory cells, and cytokine secretion [1, 2, 3, 4], all of which are yet to be explored in conjunction with nanopore stimulation with potential implications for the future development of B cell‐based immunotherapies. We have provided a morphological assessment, particularly on the state of B cell microvilli that extend into nanopores, at 30 min after plating, which should be expanded by 24 h and an even longer time point (e.g. 3–5 days) to capture potential dynamic changes in cellular morphology and/or pBTK/pZAP70/BCR immunolabeling profiles. Similarly, future flow cytometric analyses should more comprehensively investigate putative dynamic changes in activation marker expression at early (several hours) and late time points (> 5 days). Besides CD69, a formally recognized early activation marker [54], this should include further early and late activation markers such as CD80 and CD83 in conjunction with testing whether their expression is linked to T cell help ([55, 56]; but see Figure S1D) as it may very well be that nanoporous substrates only achieve partial B cell activation and that full as well as sustained B cell activation would then be reliant on external factors such as helper T cells.

Actin cytoskeleton remodeling typically occurs following BCR‐mediated B cell stimulation and appears to be both a permissive and instructive component of B cell activation [31, 40, 41, 57]. This underlies cellular changes including immunological synapse formation with APCs, antigen interrogation via the BCR, and confinement of BCR lateral diffusion within the synapse to boost signaling downstream of the BCR. Actin cytoskeleton remodeling and actin filament formation were also observed in our nanopore‐mediated B cell activation paradigm. However, rather than being a cell‐wide phenomenon like on positive control substrates that cross‐link BCR and ligate CD40, F‐actin exhibited distinct accumulations within microvilli protrusions into nanopores, establishing a potential mechanistic link between actin‐based microvilli formation into nanopores and B cell activation. Of note, T cell microvilli have previously been reported to protrude into the plasma membrane invaginations on APCs, where they create signaling hotspots leading to activation [28, 58]. This may underscore the utility of our nanoporous substrate as a platform to model lymphocyte‐APC interactions within the context of lymphocyte activation. Indeed, our nanopore‐invading B cell microvilli contained phosphorylated proteins typically associated with signaling downstream of the BCR (pBTK, pZAP70), which is indicative of B cell activation. However, they were frequently devoid of the IgM variant of the BCR itself (Figure 1C). By contrast, T cells that similarly extend actin‐rich microvilli into nanopores, also exhibit TCR accumulation within microvilli, the clustering of which was hypothesized to be responsible for nanopore‐mediated T cell activation [27]. Whether and in what capacity the BCR, including smaller isoforms thereof, is involved in nanopore‐mediated B cell activation remains to be elucidated. In further support of a mechanistic link between actin remodeling and B cell activation, B cell treatment with Cytochalasin D increased both pBTK presence within microvilli and CD69 cell surface expression. This may conceivably be due to a consequent increase of intracellular Ca^2+^ [46, 47, 48, 59], which is an integral component behind B cell activation [17, 35], as was confirmed by our Ca^2+^ level manipulation experiments (Figure 6B–D). Although Cytochalasin D is generally recognized as an actin filament destabilizing drug, there are reports of Cytochalasin D promoting filament polymerization, at least in murine B cells [47]. This could explain the emergence of longer microvilli structures in our experiments. However, why and how Jasplakinolide treatment, formally known to induce actin filament polymerization [60, 61], failed to induce microvilli formation and BTK phosphorylation in our hands (Figure 5L–O), yet stimulated high levels of CD69 activation marker expression on the cell surface (Figure 6F), remains to be investigated. Nonetheless, the fact that pharmacological actin filament manipulation by both stabilization and destabilization can activate B cells in the absence of providing a BCR‐ligating stimulus is in keeping with previously published findings [46, 47, 48, 59]. The authors of [59] provide evidence that BCR signaling is initiated indirectly without canonical ligation by increasing the BCR membrane diffusion coefficient, which may either increase encounters with coreceptors or activated signaling components to amplify BCR's inherent tonic signaling capacity or promote BCR segregation from inhibiting phosphatases such as CD45. In any case, such actin manipulations then engage typical signaling components downstream of the BCR (LYN, BLNK, BTK, and PLCγ2), eventually producing measurable B cell activation readouts such as intracellular Ca^2+^ spikes and phosphorylation of known signaling molecules such as ERK.

Our study sought to discover the mechanism(s) exclusively guiding nanopore‐mediated B cell activation. This criterion was fulfilled by MSCs, the inhibition of which using GsMTx‐4 specifically attenuated activation by nanopores but not activation via a conventional stimulation paradigm reliant on BCR cross‐linking and CD40 ligation. While we note that these pharmacological findings need to be confirmed by future genetic studies, this is in contradiction with a previously published report, in which activation via BCR cross‐linking was sensitive to GsMTx‐4 [23]. Nonetheless, our nanopore‐based findings can be embedded within an existing mechanistic framework that is centered around Ca^2+^ as a key regulator of B cell activation. Here, we speculate whether the conceivably extreme plasma membrane curvatures achieved at the tip and base of microvilli were sufficient to cause Ca^2+^ influx through MSCs. Conventionally, however, BCR engagement triggers a two‐step increase in intracellular Ca^2+^ that requires PLCγ‐mediated Ca^2+^ release from ER stores and Ca^2+^ influx from the extracellular space through CRAC channels (SOCE). It is this SOCE‐derived Ca^2+^ that is crucial for lymphocyte responses to stimuli as evidenced by the existence of immunodeficiency disorders associated with dysfunctional SOCE [62, 63, 64]. For T cells, nanopore‐induced stabilization of microvilli promotes TCR clustering within microvilli and signal initiation via Ca^2+^ potentially driven by close contact formation [27, 52] and possibly also MSC involvement via altered membrane curvature and tension. In B cells, however, MSC‐mediated Ca^2+^ entry following nanopore stimulation potentially bypasses BCR engagement‐dependent Ca^2+^ release from ER stores. This is plausibly supported by the differing Ca^2+^ spike profiles in our study between BCR/CD40‐ and nanopore‐stimulated B cells. Nonetheless, putative interactions in B cells between MSCs and ER‐resident components of Ca^2+^ homeostasis should be addressed in future studies based on published findings [59, 65]. Whether and to what extent SOCE is involved in intracellular Ca^2+^ level increases in nanopore‐stimulated B cells is also yet to be explored. At least in endometrial stem cells, inhibiting SOCE partially attenuated Ca^2+^ currents through the MSC member PIEZO1 [66], indicative of a bridging mechanistic link. The exact nature of interaction between nanopore‐mediated Ca^2+^ signaling and other B cell activation signaling components (e.g., BTK, CD69) should also be addressed in future studies. What is presently known is that overexpression of BTK on its own leads to SOCE by stimulating Ca^2+^ store depletion and influx of extracellular Ca^2+^ following BCR crosslinking in B cell lines [38]. Vice versa, while Ca^2+^ chelation with BAPTA‐AM depressed pBTK levels, arguing in favor of a feedback mechanism between BTK and intracellular Ca^2+^, pBTK levels remained unchanged following GsMTx‐4‐mediated Ca^2+^ influx inhibition via MSCs. We can presently only speculate if this could have been achieved using higher GsMTx‐4 concentrations or if Ca^2+^ influx through MSCs bypasses BTK as a signaling hub. It is furthermore entirely possible that the perhaps relatively nuanced changes in pBTK levels require more sensitive pBTK detection methods such as Western blot, ELISA, and phosphor flow.

At a physiological level, we can presently only speculate what the in vivo pendant to our in vitro B cell activation model may be. Likely scenarios include instances where developing and/or maturing B cells interact with the bone marrow or spleen environment to guide their differentiation [67, 68]. Circulating B cells that have extravasated into surrounding tissues in cases of injury or disease likely also experience the extracellular matrix (ECM) within an inflammatory context, i.e., potentially denser than usual (ECM fibrosis), much like B cells in swollen lymph nodes experience a different mechanobiological context during infection or illness compared to when one is healthy. In cancer biology, the tumor microenvironment is known to be a milieu where different mechanobiological phenomena (e.g., tissue stiffness, solid/fluid/shear stress, and ECM topology as well as pore characteristics) modulate the disease [69, 70, 71], while the presence of tumor‐infiltrating B cells can influence disease progression [72]. Opportunities for B cells to probe their environment for mechanical input via microvilli are thus ample, and future studies should investigate whether and how physiological interactions between B cell microvilli and “natural nanopores” guide B cell behavior. This in turn may create promising new avenues for future therapies, thus expanding the existing repertoire of B cell‐based and/or B cell‐targeting immunotherapies [22, 73]. The nanoporous substrate platform developed by our group could be a useful tool in this endeavor both as a B cell interaction model that is amenable to customization according to model needs and as a vehicle for the generation of B cell‐based therapies where activation is required.

## 4 Conclusion

Our findings demonstrate that mechanobiological stimulation via nanoporous substrates leads to B cell activation, as has previously been demonstrated by our group to be the case for T cells [27]. Activation, albeit relatively moderate in extent, appears to be linked to microvilli extension into nanopores and general intracellular aspects of B cell activation like actin cytoskeleton remodeling and Ca^2+^ signaling, which would normally occur downstream of BCR‐mediated activation. However, the relative absence of BCR from microvilli suggests that the involvement of these activation components may by BCR‐independent. Rather, we discovered MSC engagement‐mediated Ca^2+^ entry as a mechanism that at least partially underlies nanopore‐mediated B cell activation, while being absent during activation paradigms that engage the BCR. As such, MSC activity may form part of a broader mechanosensitive feedback mechanism linking cytoskeletal dynamics, microvillar architecture, and Ca^2+^ homeostasis. Within a physiological setting between B cells and APCs, this may speculatively provide modulating activation input on top of BCR‐dependent stimulation. While further studies are needed, our findings have potential implications for modeling interactions between B cells and their environment as well as the development of B cell‐reliant immunotherapies.

## 5 Experimental Section

### 5.1 Contact for Reagent and Resource Sharing

Further information and requests for resources and reagents should be directed to and will be fulfilled by the lead contacts, Dominic Aghaizu and Enrico Klotzsch (dominic.aghaizu@hu-berlin.de, enrico.klotzsch@charite.de).

### 5.2 Experimental Model and Subject Details

The study was approved by the Charité‐Universitätsmedizin Berlin Ethics Committee, and peripheral blood was obtained from healthy donors who had given their written informed consent (Ethics Committee of the Charité approval EA4/091/19). A total of 18 separate healthy donors participated in this study. All experiments were performed in accordance with relevant guidelines and regulations.

### 5.3 Method Details

#### 5.3.1 Cell Culture Substrate Preparation

Appropriately dimensioned cell culture substrates able to fit into cell culture multiwell plates were cut from nanoporous aluminium sheets (FlexiPor, pore depth ∼5–10 µm, pore diameter ∼260 nm; Smartmembranes) or control flat aluminium sheets (thickness 200 µm; Smartmembranes) using a 60 W split fiber laser engraver (Omtech) with the following settings: speed 100 mm/s, max. power 90%, frequency 100 kHz, Q pulse width 200 ns. Cut‐out substrates were subsequently placed in suitable cell culture multiwell plates and washed with 0.1% Tween‐20 (BioRad) in ddH_2_O for 15 min at room temperature (RT) while placed on a shaker. Substrates were then washed once with ddH_2_O, followed by a 70% ethanol wash step for 15 min at RT on a shaker. The ethanol was then replaced with 100% isopropanol for an additional wash for 15 min at RT on a shaker. Following isopropanol removal, substrates were allowed to dry overnight. Substrates were then treated in an air plasma cleaner (6 min, at 18 W, using a Plasma cleaner Zepto model 2; Diener) and then immediately covered with 25 mM MES, 0.05% Tween‐20 in ddH2O (pH 5), additionally containing 10 µg/mL neutravidin (ThermoFisher) if subject to antibody coating. Following incubation for 30 min at RT on a shaker, substrates were washed 3× with PBS (VWR), after which either PBS or, if subject to antibody coating, biotinylated coating antibodies for B cell activation were applied at 10 µg/mL in PBS for 30 min at RT on a shaker (mouse anti human IgM, Southern Biotech, 9022‐08, RRID:AB_2796585; mouse anti human CD40, Biolegend, 334343, RRID:AB_2566580). Substrates were subsequently washed 3× with PBS and treated with UV light for 20 min inside a laminar flow hood for sterilization while covered with PBS. PBS was then replaced with B cell culture medium (RPMI 1640 (PAN Biotech) supplemented with 10% FBS (ATCC‐LGC Standards), 2 mM L‐Glu, 20 mM HEPES, 1× penicillin‐streptomycin (ThermoFisher)) for pre‐equilibration at 37°C/5% CO_2_ until cell plating.

#### 5.3.2 PBMC Isolation

Freshly obtained buffy coat ordered from Deutsches Rotes Kreuz was first diluted 1:4 with Ca^2+^‐free PBS (VWR) at room temperature. For each 50 mL SepMate tube (Stemcell Technologies), 35 mL of diluted buffy coat was gently layered on top of 15 mL Lymphoprep density gradient medium (Stemcell Technologies) previously added to the SepMate tube. The samples were centrifuged at 1200 g for 20 min at room temperature. The PBMC‐containing layer was subsequently transferred to fresh 50 mL tubes, and cells were pelleted at 120 g for 10 min. Following supernatant removal, PBMCs were washed with Ca^2+^‐free PBS and centrifuged at 300 g for 10 min. Pelleted PBMCs were resuspended with FBS supplemented with 10% DMSO at a concentration of 1 × 10^8^ cells/mL, aliquoted typically at 5 × 10^7^ cells per freezer vial, and stored overnight inside a Mr. Frosty freezing container at ‐80°C prior to long‐term storage in liquid nitrogen.

#### 5.3.3 B Cell Isolation

Primary resting human B cells were purified from frozen PBMC preparations by negative selection using the B cell isolation kit II (Miltenyi Biotec) according to the manufacturer's instructions. Briefly, frozen PBMCs were thawed in a 37°C water bath until mostly liquid. PBMCs were transferred to 15 mL tubes, which was subsequently completely filled by dropwise addition with cold MACS buffer (PBS supplemented with 0.5% w/v BSA (Sigma), 2 mM EDTA (Life Technologies)). Following centrifugation at 400 g, 4°C for 10 min, the PBMC pellet was resuspended in 30 µL cold MACS buffer and 10 µL Biotin‐antibody cocktail per 1 × 10^7^ cells and incubated at 4°C for 5 min. Hereafter, a further 30 µL cold MACS buffer and 20 µL anti‐Biotin Microbeads per 1 × 10^7^ cells were added to the sample, followed by incubation at 4°C for 10 min. During incubation, an LS MACS column (Miltenyi Biotec) fitted with a 30 µm pre‐separation filter (Miltenyi Biotec) was placed in the magnetic field of a Midi MACS separator (Miltenyi Biotec) and washed with 3 mL MACS buffer. The cell suspension was then applied to the filter stacked on top of the pre‐equilibrated MACS column, and the flow‐through containing unlabeled/untouched primary resting B cells was collected in 15 mL tubes. The filter‐LS column assembly was subsequently washed 3× with 1 mL MACS buffer with continued collection of flow‐through, followed by cell yield determination in flow‐through. Enriched B cells pelleted by centrifugation at 400 g for 10 min were then resuspended in an appropriate volume of B cell culture medium (RPMI 1640 (PAN Biotech) supplemented with 10% FBS (ATCC‐LGC Standards), 2 mM L‐Glu, 20 mM HEPES, 1× penicillin‐streptomycin (VWR)) and incubated at 37°C/5% CO_2_ until use.

#### 5.3.4 Cell Culture and Pharmacology

For experiments with flow cytometry readout, isolated B cells were seeded on previously prepared and preequilibrated substrates in multiwell plates at a cell density of 2.7 × 10^5^ cells/cm^2^ diluted in B cell culture medium (RPMI 1640 (PAN Biotech) supplemented with 10% FBS (ATCC‐LGC Standards), 2 mM L‐Glu, 20 mM HEPES, 1x penicillin‐streptomycin (VWR)) and incubated at 37°C/5% CO_2_ for 24 h until harvesting.

For experiments with confocal microscopy readout, isolated B cells were seeded on previously prepared and preequilibrated substrates in multiwell plates at a cell density of 1.4 × 10^5^ cells/cm^2^ diluted in B cell culture medium. B cells were then subjected to spin plating in a centrifuge at 50 g for 5 min at RT. B cells were subsequently incubated at 37°C/5% CO_2_ for 30 min until fixation.

For pharmacological studies, isolated B cells were additionally pre‐incubated at suitable cell densities with pharmacological agents at 37°C/5% CO_2_ for 1 h prior to plating. Pharmacological agents used in this study: 10 µM BAPTA‐AM (Fisher Scientific), 10 µM Cytochalasin D, 10 µM GsMTx‐4 (Hölzel Biotech), 10 nM Ibrutinib (Fisher Scientific), 1 µM Ionomycin (Sigma), 2 µM Jasplakinolide (Sigma; kind gift from Dr. Bär), and 1 µM Thapsigargin (Fisher Scientific; kind gift from Dr. Bertelli).

#### 5.3.5 Generation of Monocyte‐Derived Dendritic Cells (moDCs)

For the generation of monocyte‐derived dendritic cells (moDCs), CD14^+^ monocytes were first isolated from frozen PBMC preparations by positive selection using CD14^+^ microbeads (Miltenyi Biotec). Briefly, frozen PBMCs were thawed in a 37°C water bath until mostly liquid. PBMCs were transferred to 15 mL tubes, which was subsequently completely filled by dropwise addition with cold MACS buffer (PBS supplemented with 0.5% w/v BSA (Sigma), 2 mM EDTA (VWR)). Following centrifugation at 300 g, 4°C for 10 min, the PBMC pellet was resuspended in 80 µL cold MACS buffer and 20 µl CD14 microbeads per 1 × 10^7^ cells and incubated at 4°C for 15 min. Cells were subsequently washed with MACS buffer, pelleted at 300 g, 4°C for 10 min, and then resuspended with 500 µL MACS buffer. An LS MACS column (Miltenyi Biotec) was placed in the magnetic field of a Midi MACS separator (Miltenyi Biotec) and washed with 3 mL MACS buffer. The cell suspension was then applied to the pre‐equilibrated MACS column and the flow‐through containing unwanted cells was collected in 15 mL tubes. The column was subsequently washed 3× with 1 mL MACS buffer with continued collection of flow‐through. The magnetically labeled CD14^+^ monocytes were then flushed out from the column by applying 1 mL MACS buffer to the column followed immediately by pushing the plunger into the column while collecting the eluent in a fresh 15 mL tube. Cells were counted and cultured in an appropriate volume of moDC differentiation medium (X‐Vivo 15 media (Lonza) supplemented with 800 U/mL GM‐CSF (PeproTech) and 250 U/mL IL‐4 (PeproTech)). Incubation at 37°C/5% CO_2_ for 6 days resulted in the differentiation of CD14^+^ monocytes into moDCs. Maturation of moDCs was induced for an additional 24 h with 1000 IU/mL TNF‐α (PeproTech), 200 IU/mL IL‐1β (PeproTech), 1000 IU/mL IL‐6 (Miltenyi Biotech), and 1 µg/mLl prostaglandin E2 (PGE2) (Enzo), resulting in mDCs.

#### 5.3.6 Immunocytochemistry

B Cells and moDCs were fixed with 4% PFA (Electron Microscopy Sciences) for 10 min at 37°C by gently dispensing pre‐warmed 8% PFA in cytoskeletal buffer (10 mM MES, 150 mM NaCl, 5 mM Glucose, 5 mM EGTA, 5 mM MgCl in ddH_2_O, pH 6.1) on top of an equal amount of cell culture volume. Fixed B cells were subsequently washed 3× with PBS and incubated with 0.01% (w/v) NH_4_Cl (Sigma) in PBS for 10 min at RT to quench autofluorescence. B cells were then washed 3× with PBS and blocked/permeabilized with 1× PBS supplemented with 5% (w/v) normal goat serum (Biolegend), 1% (w/v) BSA (Sigma), and 0.01% (v/v) Triton x‐100 (Sigma) for 1 h at RT. Primary antibodies were diluted in blocking solution (1× PBS supplemented with 5% (w/v) normal goat serum (Biolegend), 1% (w/v) BSA (Sigma)) and applied to B cell samples at 4°C overnight following removal of blocking/permeabilization buffer. Primary antibodies used in this study were: beta actin (Synaptic Systems, 251006, RRID:AB_2782985, 1:500), CD45 (Abcam, ab30470, RRID:AB_726544, 1:200), pBTK (Fisher Scientific, NBP1‐78295, RRID: AB_11032627, 1:200), moesin (Abcam, ab52490, RRID:AB_881245, 1:200) and pZAP70 (Cell Signaling, 2717S, RRID:AB_2218658, 1:250). B cells were then washed 3× for 5 min each with PBS and subsequently incubated with secondary antibodies, antibody‐dye conjugates and/or labeling dyes diluted in blocking solution for 2 h at RT in the dark. Reagents used were: anti human HLA‐DR PE (Miltenyi, 130‐111‐942, RRID:AB_2726058, 1:50), goat anti mouse IgG Dylight 405 (ThermoFisher, 35500BID, RRID:AB_2533208, 1:250), goat anti human IgM Alexa Fluor 488 (Jackson ImmunoResearch, 109‐547‐043, RRID: AB_2337855, 1:400), goat anti rabbit IgG Alexa Fluor 568 (ThermoFisher, A‐11036, RRID: AB_10563566, 1:500), and phalloidin Alexa Fluor 647 (ThermoFisher, A22287, 1:300). B cells were then washed 3× for 5 min each with PBS at RT in the dark, and samples were mounted using Prolong gold antifade reagent (Molecular Probes).

### 5.4 Microscopy

#### 5.4.1 Live Cell Ca^2+^ Time Lapse Microscopy

All experimental runs were carried out with freshly isolated primary resting human B cells (see Methods; B cell isolation). B cells were washed with Hank's balanced salt solution (HBSS; PAN Biotech) buffer by filling the tube with buffer. Cells were subsequently pelleted at 500 g for 5 min at RT, resuspended in HBSS supplemented with intracellular Ca^2+^ indicator Fluo4‐AM (Invitrogen; 4.5 µM) at 1 × 10^6^ cells/mL, and incubated at RT for 1 h in the dark. B cells were washed once more with HBSS, pelleted at 500 g for 5 min at RT, and resuspended in B cell culture medium (without phenol red) at a cell density of 1 × 10^6^ cells/ml. 1 × 10^5^ cells were plated on previously prepared cell culture substrates (flat, flat (αCD40, αIgM) or nanoporous substrate) placed in custom 3D printed PLA chambers, pre‐incubated at 37°C for 30 min filled with B cell culture medium, and covered with a round coverslip before immediately proceeding to live cell Ca^2+^ imaging to acquire time lapse recordings from the time of contact establishment with the substrate onward.

Live cell Ca^2+^ imaging was carried out on an upright Leitz Laborlux S microscope (Leitz) equipped with an HBO lamp, a 10× objective (Nikon), an Andor Clara EMCCD897 camera, and a custom environmental chamber set to 37°C using an Ibidi heating system. A GFP excitation filter set (525/50 bandpass) was used for the isolation of Fluo‐4‐AM fluorescence emission. 30 min time‐lapse live Ca^2+^ recordings were acquired with 1 s integration time and 1 frame per second with 2 × 2 binning, resulting in a resolution of 696 × 520 pixels and a pixel size of 12.9 × 12.9 µm. All steps were performed without CO_2_ control.

#### 5.4.2 Spinning Disc Confocal Microscopy of Fixed Specimens

Fixed and stained B cells and moDCs were imaged using an Olympus FV1000‐based spinning disc confocal microscope assembled by Visitron, fitted with a 150x (NA = 1.15) objective and an Andor iXon Ultra 888 EMCCD camera to detect fluorescence emission. Dylight 405, Alexa Fluor 488, Alexa Fluor 568, and Alexa Fluor 647 fluorophores were excited using 405, 488, 561, and 647 nm lasers, respectively. For image acquisition, *xyz* confocal stacks were captured at a resolution of 1024 × 1024 pixels and at a step size of 0.15 µm, resulting in voxel dimensions of 0.0867 × 0.0867 × 0.15 µm. Microscope settings were established during the first acquisition and subsequently not further modified.

#### 5.4.3 Flow Cytometry With Sample Preparation

Following incubation at 37°C/5% CO_2_ for 24 h, B cells were harvested, transferred to 96‐well V‐bottom plates, and pelleted at 500 g for 5 min. Cells were subsequently washed with 200 µL MACS buffer (1× PBS supplemented with 0.5% BSA and 2 mM EDTA) and pelleted once more at 500 g for 5 min. Conjugated antibodies for flow cytometry alongside FC block (Miltenyi, 1:50) were diluted in MACS buffer and applied to B cell samples at 4°C for 30 min in the dark. Antibodies used in this study were: human CD3‐PE (clone OKT3, Biolegend, 317308, RRID:AB_571913, 1:100), human CD19‐BV421 (clone SJ25C1, Biolegend, 363018, RRID:AB_2564227, 1:75), human CD19‐AF647 (clone HIB19, Biolegend, 302220, RRID:AB_389335, 1:75), human CD69‐APC (clone FN50, Biolegend, 310910, RRID:AB_314845, 1:100), human CD80‐BV421 (clone 2D10, Biolegend, 305222, RRID:AB_2564407, 1:100), human CD80‐PE/Dazzle594 (clone 2D10, Biolegend, 305230, RRID:AB_2566489, 1:100), human CD83‐APC/Vio770 (clone REA714 | HB15, Miltenyi, 130‐110‐506, RRID: AB_2659318, 1:200), human CD83‐FITC (clone HB15e, Biolegend, 305306, RRID:AB_314514, 1:100), human CD86‐BV650 (clone IT2.2, Biolegend, 305428, RRID:AB_2563823, 1:100), human HLA‐DR‐BV711 (clone L243, Biolegend, 307644, RRID:AB_2562913, 1:100), human IgD‐BV605 (clone IA6‐2, Biolegend, 348232, RRID:AB_2563337, 1:100), human IgG‐PE/Cy7 (clone M1310G05, Biolegend, 410722, RRID:AB_2750227, 1:100) and human IgM‐BV421 (clone MHM‐88, Biolegend, 314516, RRID:AB_2561443, 1:100). B cells were subsequently pelleted at 500 g for 5 min, washed with 200 µL PBS, and subjected to live/dead staining using Zombie NIR reagent (Biolegend) diluted 1:1000 in PBS or Ghost Dye Violet 510 (Cell Signaling) diluted 1:5000. Following incubation at 4°C for 30 min in the dark, cells were twice spun down at 500 g for 5 min and washed with 200 µL 3% BSA in PBS. B cells were then fixed with 4% PFA in PBS for 10 min at RT and, after a last centrifugation step at 500 g for 5 min, finally resuspended in 250 µL MACS buffer. Stained samples were kept at 4°C until ready for flow analysis.

Samples were analyzed on BD FACS Aria II and SonyID7000 flow cytometers with appropriately stained control samples for compensation correction. Data analysis was carried out using Flowjo v10.10.0 software (Treestar).

#### 5.4.4 Analysis of Live Cell Ca^2±^ Time Lapse Microscopy Data

Time‐lapse live Ca^2+^ recordings were imported into Fiji/ImageJ. All images were background corrected by applying a rolling ball filter with a 20 pixel radius and subsequently analyzed using Trackmate [74, 75]. Subpixel localization was set to “on” and cell diameter was estimated at 10 pixels. Fluo4‐AM‐labeled B cells and their signal intensities were detected in each frame using the Trackmate LoG spot detector in Fiji/ImageJ and spots were linked in time‐lapse tracks using the LAP tracker with the following settings: linking distance, 3 pixel; gap closing distance, 3 pixel; max frame gap, 1 frame. Tracks were filtered by their start frame, excluding cells that were already on the surface in the first frame of the measurement to be able to synchronize with respect to contact establishment with the substrate. In addition, tracks with less than 200 time points were excluded due to an insufficient number of data points.

B cell Fluo4‐AM fluorescence intensity tracks were subsequently exported into a custom‐written script in Python. Time‐lapse intensity profiles were pre‐processed using a Savitzky‐Golly‐Filter (window length, 51; polyorder, 2) for smoothing, and the first 25 frames were cut off to exclude frames before contact establishment with substrate. B cell signal intensity was normalized by the intensity at the resulting first time point (t = 0), resulting in time points stated as fold change arbitrary intensity unit values with respect to the intensity at t = 0. Fluo4‐AM Ca^2+^ spikes were detected by applying filters for spike height detection from intensity profiles (threshold, 100 AU) and spike gradient detection from corresponding first derivative curves (threshold, 0.6), similar to the approach of CalQuo software [76]. Based on Fluo4‐AM Ca^2+^ intensity profiles, B cells were classified into cells exhibiting 0, 1, or >1 Ca^2+^ spikes.

#### 5.4.5 Analysis of Microvilli Protrusions Into Nanoporous Substrate

For the analysis of B cell microvilli protrusions into nanoporous substrate, the F‐actin‐rich microvilli were stained with a fluorophore‐conjugated phalloidin in addition to co‐immunostaining for the IgM variant of the B cell receptor as well as a marker for B cell activation, pBTK. Acquired images were imported into Fiji/ImageJ for initial inspection and image processing/formatting suitable for subsequent steps (i.e., splitting 3 channel *xyz* image stacks into 2 × 2 channel *xyz* image stacks, respectively pairing phalloidin staining with IgM or pBTK immunolabeling).

Images were subsequently imported into a custom‐written script in Python for semi‐automated analysis of microvilli and immunolabeling signal protrusions into nanoporous substrates. Briefly, images were normalized and processed using a median filter, Gaussian filter (sigma, 1.0), and Laplacian of Gaussian filter, followed by microvilli detection within a *z* slice of a given image stack that exhibited clearly resolved phalloidin^+ve^ microvilli. Based on phalloidin staining, the following parameters were used for local maxima detection corresponding to microvilli within script‐generated cell masks: intensity threshold, 625; sigma (radius), 1.5. Microvilli coordinates within this single slice were then extended to the entire image stack and *z* intensity profiles generated for phalloidin, IgM, and pBTK signals for as long as signal intensities remained above 1.5× standard deviations of the background signal at the first slice within the image stack exhibiting microvilli (i.e., at the surface of the nanoporous substrate). Additionally, integrated density measurements for pBTK along microvilli were extracted and quantified from this dataset and normalized by cell number using a custom‐written script in Python.

#### 5.4.6 Analysis of Flow Cytometry Data

Flow cytometry data was analyzed using FlowJo Software (v10.10.0; Treestar). Raw FCS files were loaded into the FlowJo workspace, followed by compensation correction using the Compensation Wizard to account for fluorescence spillover and data generated from compensation control runs from unstained as well as single‐stained B cells. Cell populations were sequentially gated based on forward scatter (FSC) vs side scatter (SSC) (to detect lymphocytes or leukocytes), singlet cells, live cells (ZombieNIR^−ve^ or GhostDye510^−ve^), B cells (CD19^+ve^, CD3^−ve^) or moDCs (HLA‐DR^+ve^), and activated B cells (CD69^+ve^, CD80^+ve^, CD83^+ve^) or mature/activated moDCs (CD80^+ve^, CD83^+ve^, and CD86^+ve^); see also Figure 3A and Figure S3D for respective representative gating strategies for B cells and moDCs. Gates were kept constant across experimental conditions within given biological repeats. Statistics for each population were extracted and compared across conditions.

### 5.5 Quantification and Statistical Analysis

All means are stated ± standard deviation, unless otherwise specified. *N* = number of biological repeats (independent blood donors), *n* = number of cells analyzed. For qualitative and quantitative assessments, at least 3 biological repeats were used per group. We used GraphPad Prism software (GraphPad Software Inc.) for statistical analyses. D'Agostino and Pearson test was used to assess the normality of datasets. For statistical tests involving one independent variable to be compared between 2 groups, we used the unpaired *t*‐test and Mann‐Whitney test for normally and non‐normally distributed datasets, respectively. For the comparison of one independent variable between >2 groups, we used One‐way ANOVA with Dunnett's comparison test. For statistical tests involving two independent variables between >2 groups, we used two‐way ANOVA with Dunnett's multiple comparisons test; significance was accepted at *p* ≤ 0.05.

## Author Contributions

Conceptualization: N.D.A. and E.K., Methodology: N.D.A., W.W., A.P., and N.S., Validation: N.D.A., Formal analysis: N.D.A., Investigation: N.D.A. and W.W., Visualization: N.D.A., Writing – original draft: N.D.A. and E.K., Writing – review and editing: N.D.A., W.W., and E.K., Funding acquisition: E.K., Supervision: E.K.

## Funding

This work was supported by the Deutsche Forschungsgemeinschaft (KL 3278/2‐1). N.D.A. was a DFG‐funded postdoctoral research associate. E.K. further acknowledges funding through Helmholtz Association program‐oriented funding as well as by Humboldt Universität zu Berlin.

## Conflicts of Interest

The authors declare no conflicts of interest.

## Supporting information




**Supporting File 1**: advs76869‐sup‐0001‐SuppMat.docx.

**Supporting File 2**: advs76869‐sup‐0002‐FigureS1_1.pdf.

**Supporting File 3**: advs76869‐sup‐0003‐FigureS2_1.pdf.

**Supporting File 4**: advs76869‐sup‐0004‐FigureS3_1.pdf.

**Supporting File 5**: advs76869‐sup‐0005‐FigureS4.pdf.

**Supporting File 6**: advs76869‐sup‐0006‐FigureS5.pdf.

**Supporting File 7**: advs76869‐sup‐0007‐FigureS6.pdf.

## Data Availability

The data that support the findings of this study are available from the corresponding author upon reasonable request.
