# Supplementary material for: B Cells are Activated via a Nanoporous Interface that Stabilizes Microvilli and Engages Mechanosensitive Ion Channels
Source: Adv Sci (Weinh). 2026 Aug 3;13(59):e76869. doi: 10.1002/advs.76869 (PMC13430938; doi:10.1002/advs.76869)
Supplement: Supplementary file 1 [file ADVS-13-e76869-s001.pdf]

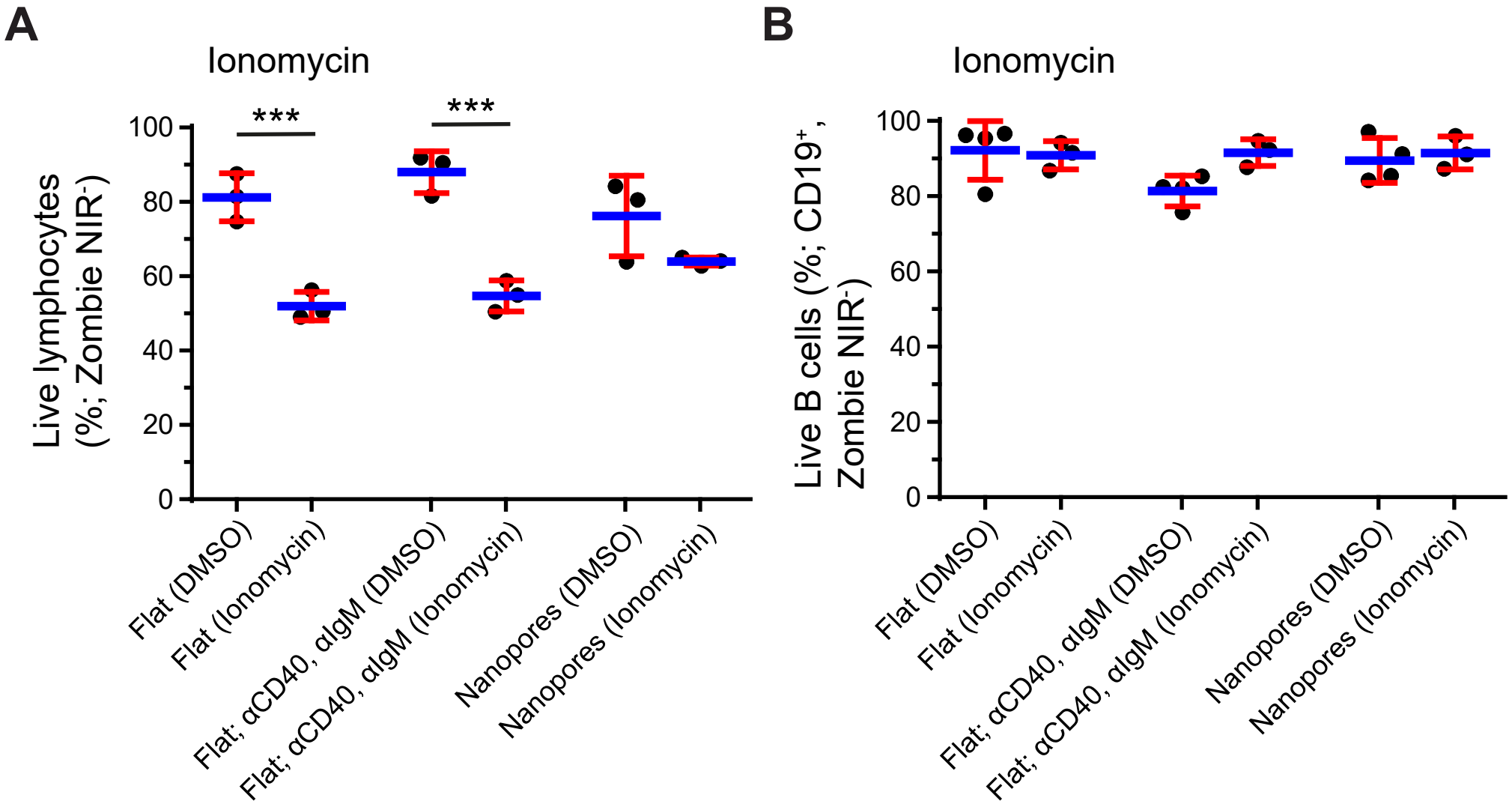

A
Ionomycin
Live lymphocytes (%; Zombie NIR-)
100
80
60
40
20
0
***
***
Flat (DMSO)
Flat (Ionomycin)
Flat; αCD40, αIgM (DMSO)
Flat; αCD40, αIgM (Ionomycin)
Nanopores (DMSO)
Nanopores (Ionomycin)
B
Ionomycin
Live B cells (%; CD19+, Zombie NIR-)
100
80
60
40
20
0
Flat (DMSO)
Flat (Ionomycin)
Flat; αCD40, αIgM (DMSO)
Flat; αCD40, αIgM (Ionomycin)
Nanopores (DMSO)
Nanopores (Ionomycin)
