# Supplementary material for: B Cells are Activated via a Nanoporous Interface that Stabilizes Microvilli and Engages Mechanosensitive Ion Channels
Source: Adv Sci (Weinh). 2026 Aug 3;13(59):e76869. doi: 10.1002/advs.76869 (PMC13430938; doi:10.1002/advs.76869)
Supplement: Supplementary file 2 [file ADVS-13-e76869-s002.pdf]

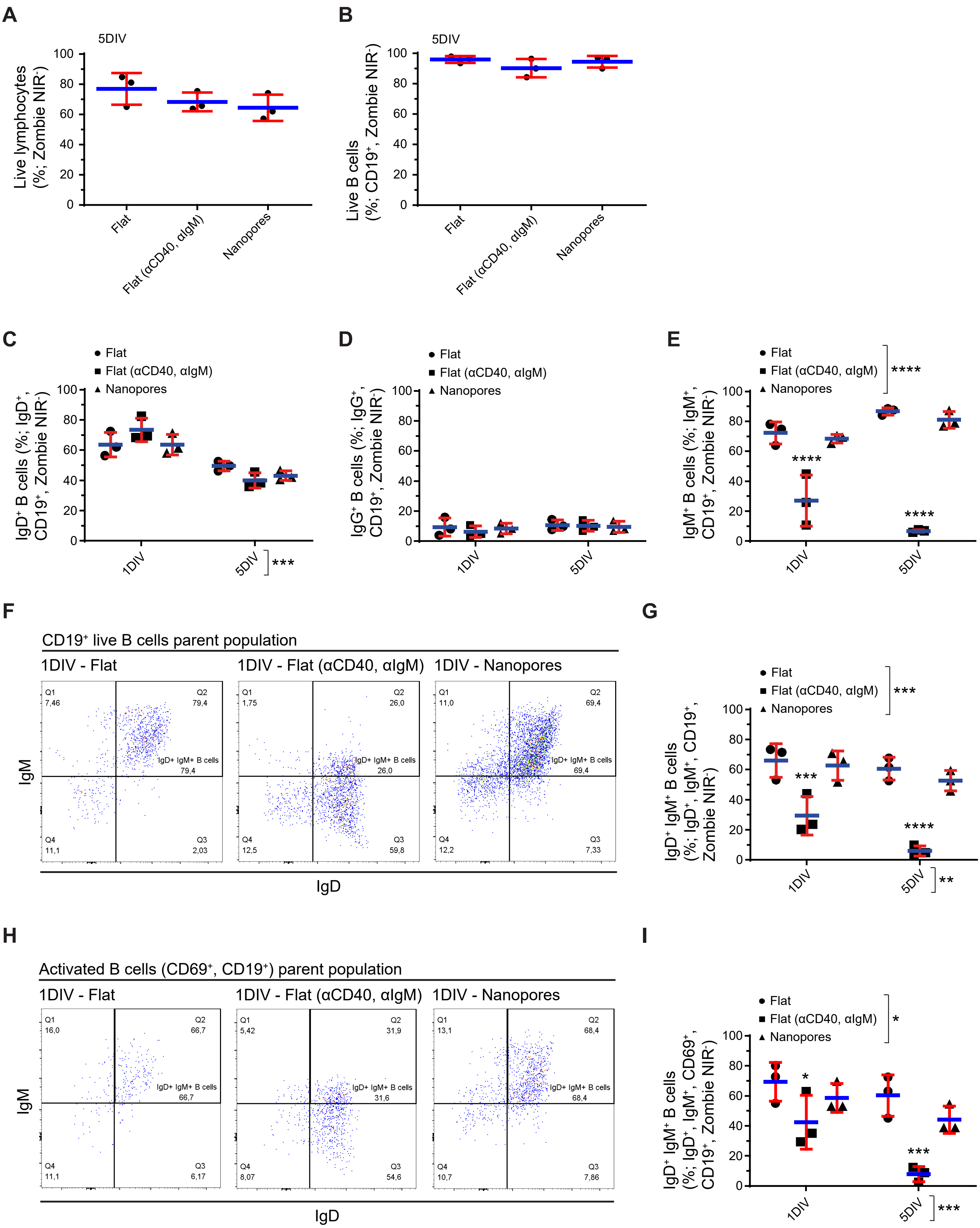
A
5DIV
Live lymphocytes (%; Zombie NIR⁻)
Flat
Flat (αCD40, αIgM)
Nanopores
B
5DIV
Live B cells (%; CD19⁺, Zombie NIR⁻)
C
IgD⁺ B cells (%; IgD⁺, CD19⁺, Zombie NIR⁻)
1DIV
5DIV
***
D
IgG⁺ B cells (%; IgG⁺, CD19⁺, Zombie NIR⁻)
E
IgM⁺ B cells (%; IgM⁺, CD19⁺, Zombie NIR⁻)
****
F
CD19⁺ live B cells parent population
1DIV - Flat
1DIV - Flat (αCD40, αIgM)
1DIV - Nanopores
IgD+ IgM+ B cells
IgM
IgD
G
IgD⁺ IgM⁺ B cells (%; IgD⁺, IgM⁺, CD19⁺, Zombie NIR⁻)
**
H
Activated B cells (CD69⁺, CD19⁺) parent population
I
IgD⁺ IgM⁺ B cells (%; IgD⁺, IgM⁺, CD69⁺, CD19⁺, Zombie NIR⁻)
*
