# Supplementary material for: B Cells are Activated via a Nanoporous Interface that Stabilizes Microvilli and Engages Mechanosensitive Ion Channels
Source: Adv Sci (Weinh). 2026 Aug 3;13(59):e76869. doi: 10.1002/advs.76869 (PMC13430938; doi:10.1002/advs.76869)
Supplement: Supplementary file 3 [file ADVS-13-e76869-s003.pdf]

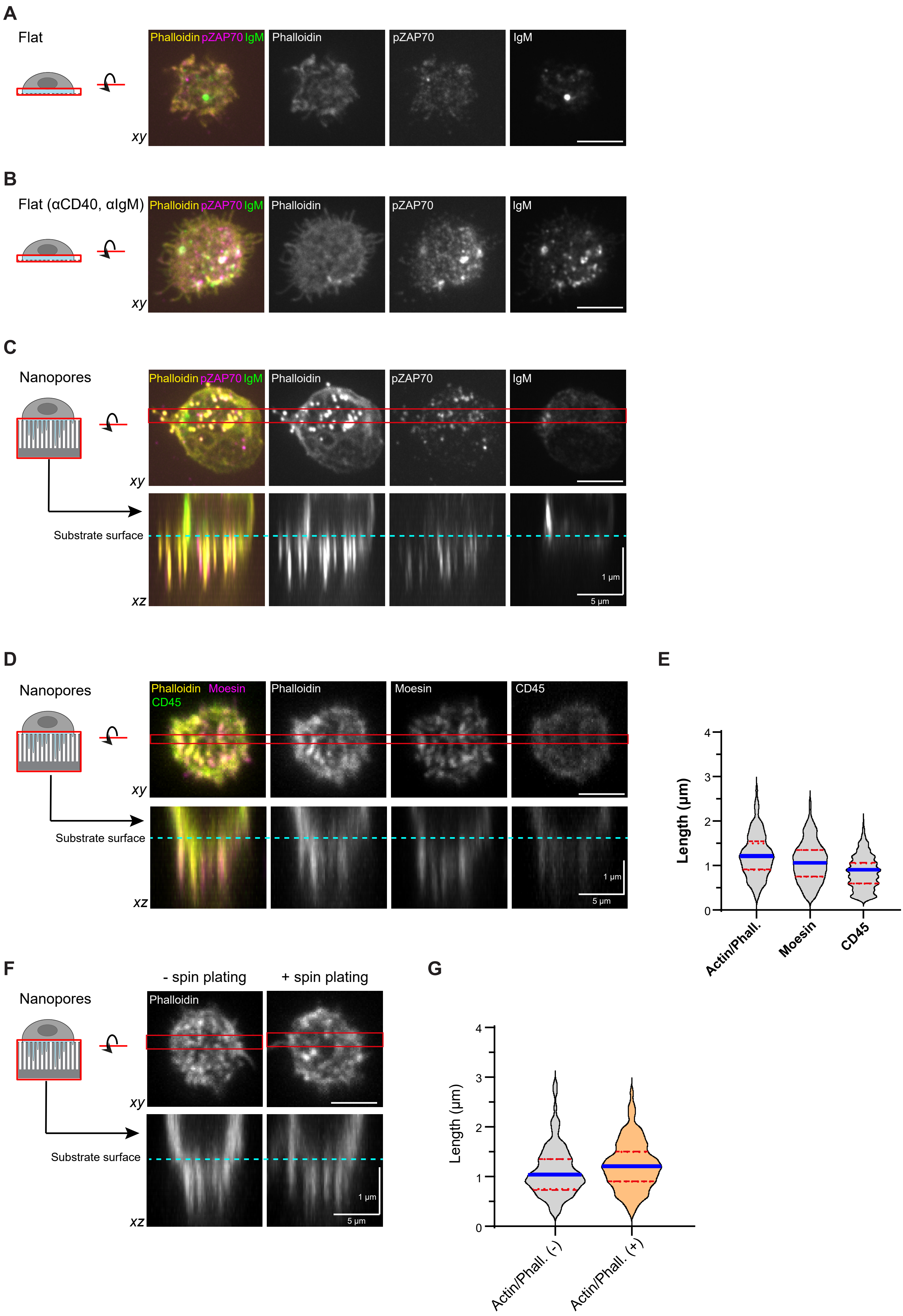

A
Flat
Phalloidin pZAP70 IgM
Phalloidin
pZAP70
IgM
xy
B
Flat (αCD40, αIgM)
Phalloidin pZAP70 IgM
Phalloidin
pZAP70
IgM
xy
C
Nanopores
Phalloidin pZAP70 IgM
Phalloidin
pZAP70
IgM
xy
Substrate surface
xz
1 µm
5 µm
D
Nanopores
Phalloidin Moesin CD45
Phalloidin
Moesin
CD45
xy
Substrate surface
xz
1 µm
5 µm
E
Length (µm)
0
1
2
3
4
Actin/Phall.
Moesin
CD45
F
Nanopores
- spin plating
+ spin plating
Phalloidin
xy
Substrate surface
xz
1 µm
5 µm
G
Length (µm)
0
1
2
3
4
Actin/Phall. (-)
Actin/Phall. (+)
