# Supplementary material for: B Cells are Activated via a Nanoporous Interface that Stabilizes Microvilli and Engages Mechanosensitive Ion Channels
Source: Adv Sci (Weinh). 2026 Aug 3;13(59):e76869. doi: 10.1002/advs.76869 (PMC13430938; doi:10.1002/advs.76869)
Supplement: Supplementary file 4 [file ADVS-13-e76869-s004.pdf]

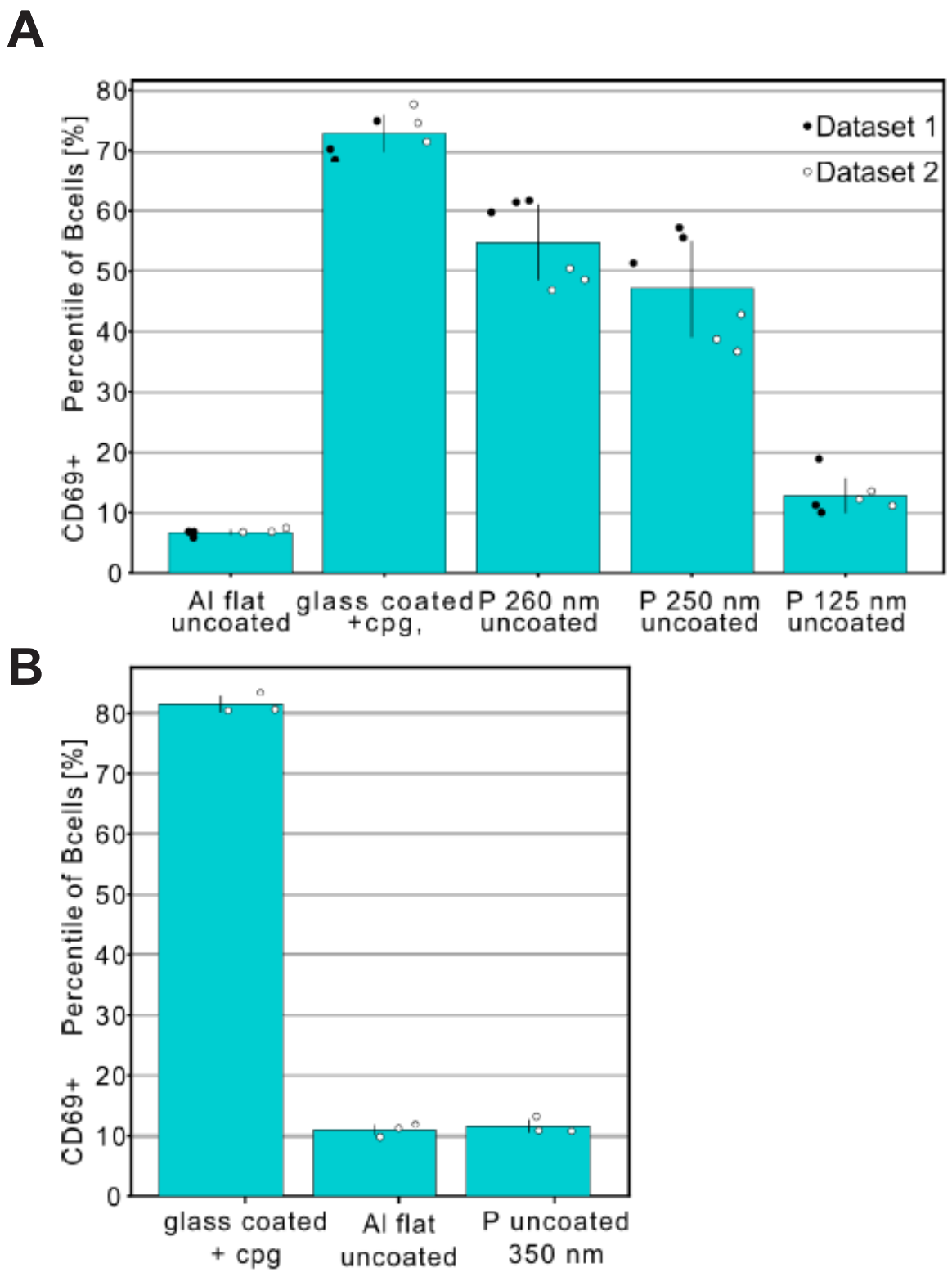
A
Dataset 1
Dataset 2
CD69+ Percentile of Bcells [%]
0
10
20
30
40
50
60
70
80
Al flat uncoated
glass coated +cpg,
P 260 nm uncoated
P 250 nm uncoated
P 125 nm uncoated
B
CD69+ Percentile of Bcells [%]
glass coated + cpg
Al flat uncoated
P uncoated 350 nm
