# Supplementary material for: B Cells are Activated via a Nanoporous Interface that Stabilizes Microvilli and Engages Mechanosensitive Ion Channels
Source: Adv Sci (Weinh). 2026 Aug 3;13(59):e76869. doi: 10.1002/advs.76869 (PMC13430938; doi:10.1002/advs.76869)
Supplement: Supplementary file 5 [file ADVS-13-e76869-s005.pdf]

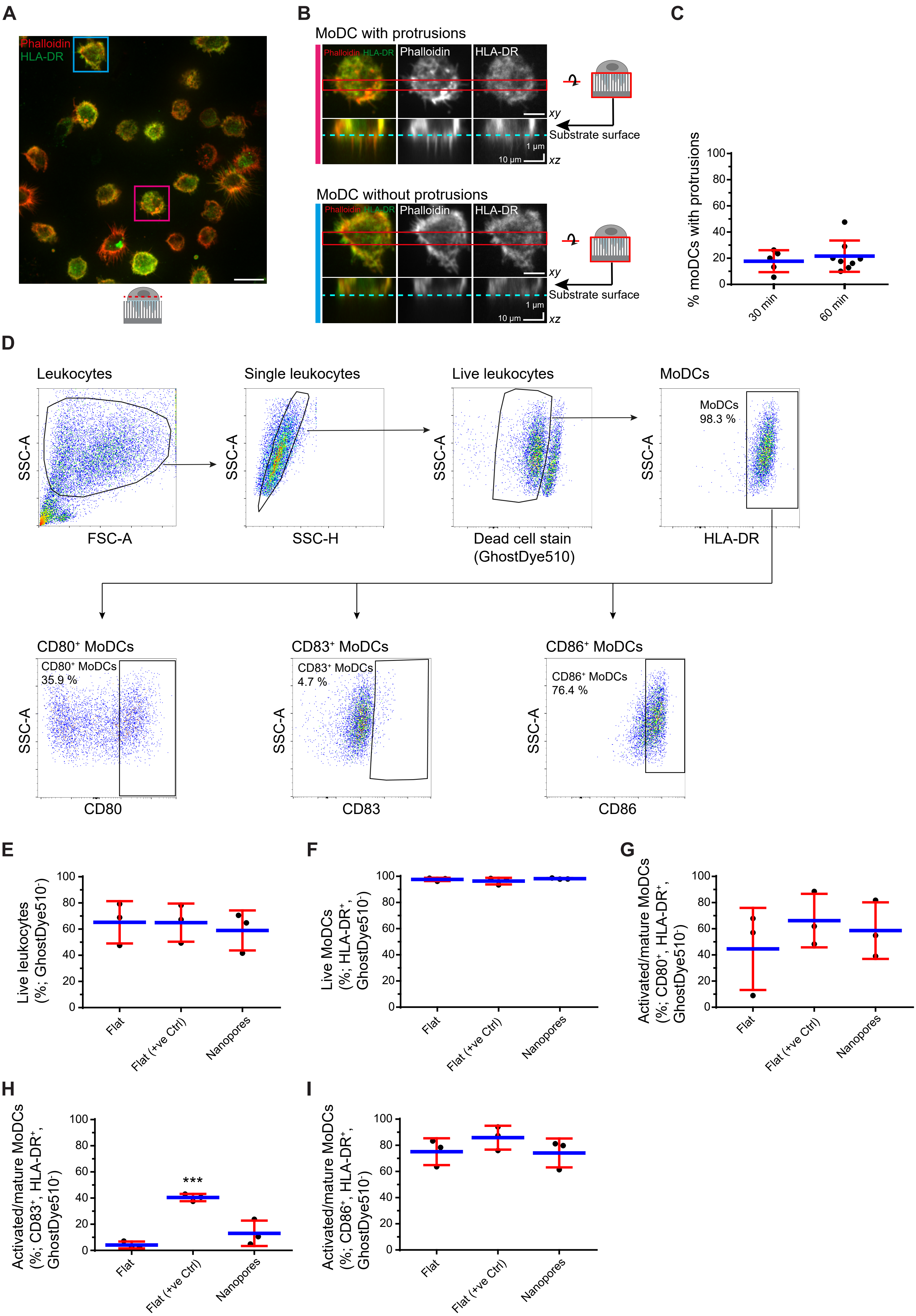
A
Phalloidin
HLA-DR
B
MoDC with protrusions
Phalloidin HLA-DR
Phalloidin
HLA-DR
xy
Substrate surface
1 µm
10 µm
xz
MoDC without protrusions
C
% moDCs with protrusions
30 min
60 min
D
Leukocytes
SSC-A
FSC-A
Single leukocytes
SSC-H
Live leukocytes
Dead cell stain (GhostDye510)
MoDCs
MoDCs 98.3 %
HLA-DR
CD80⁺ MoDCs
CD80⁺ MoDCs 35.9 %
CD80
CD83⁺ MoDCs
CD83⁺ MoDCs 4.7 %
CD83
CD86⁺ MoDCs
CD86⁺ MoDCs 76.4 %
CD86
E
Live leukocytes (%; GhostDye510⁻)
Flat
Flat (+ve Ctrl)
Nanopores
F
Live MoDCs (%; HLA-DR⁺, GhostDye510⁻)
G
Activated/mature MoDCs (%; CD80⁺, HLA-DR⁺, GhostDye510⁻)
H
Activated/mature MoDCs (%; CD83⁺, HLA-DR⁺, GhostDye510⁻)
***
I
Activated/mature MoDCs (%; CD86⁺, HLA-DR⁺, GhostDye510⁻)
