# Supplementary material for: B Cells are Activated via a Nanoporous Interface that Stabilizes Microvilli and Engages Mechanosensitive Ion Channels
Source: Adv Sci (Weinh). 2026 Aug 3;13(59):e76869. doi: 10.1002/advs.76869 (PMC13430938; doi:10.1002/advs.76869)
Supplement: Supplementary file 6 [file ADVS-13-e76869-s006.pdf]

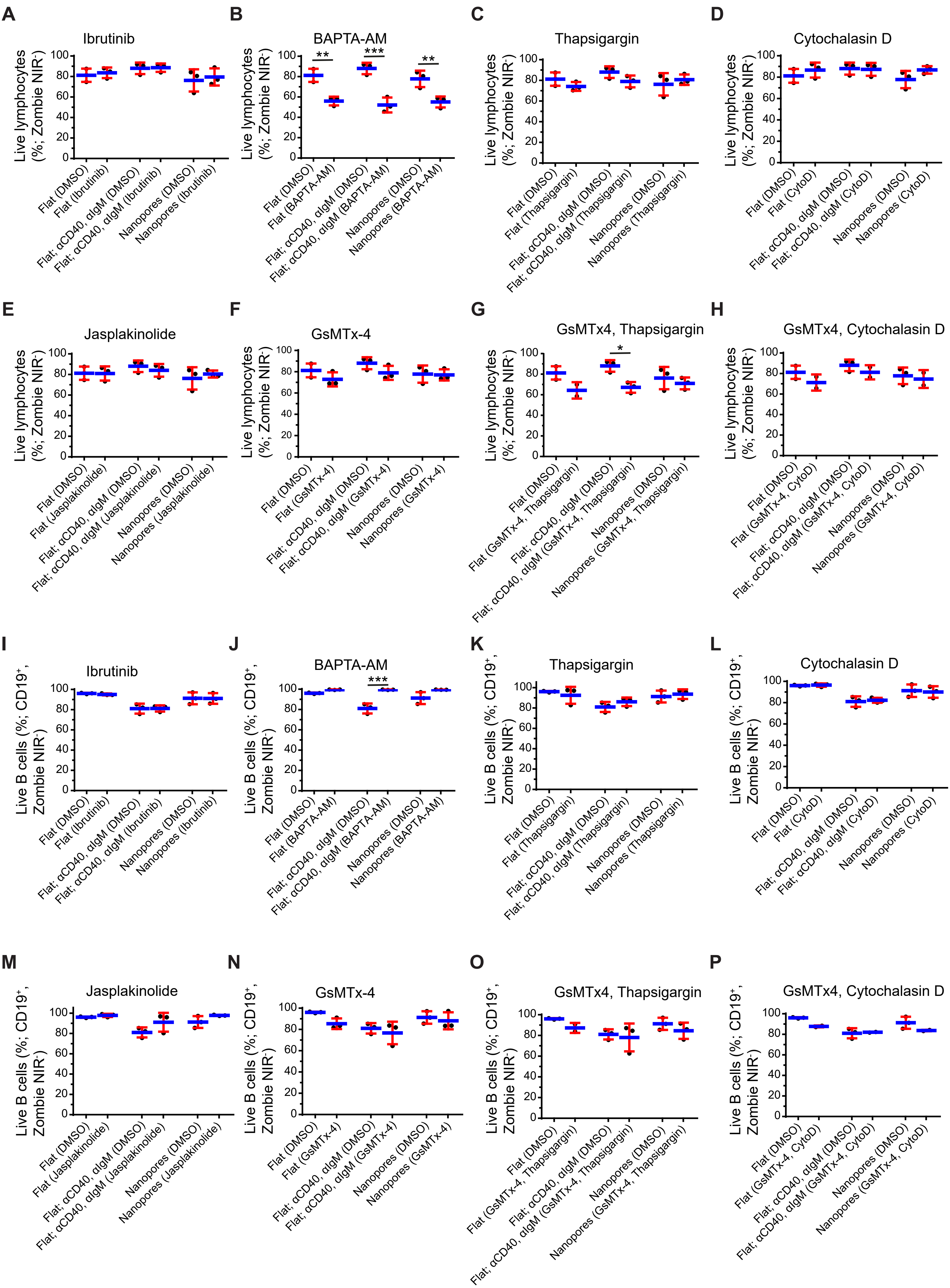

A
Ibrutinib
Live lymphocytes (%; Zombie NIR-)
Flat (DMSO)
Flat (Ibrutinib)
Flat; αCD40, αIgM (DMSO)
Flat; αCD40, αIgM (Ibrutinib)
Nanopores (DMSO)
Nanopores (Ibrutinib)
B
BAPTA-AM
Live lymphocytes (%; Zombie NIR-)
**
***
**
Flat (DMSO)
Flat (BAPTA-AM)
Flat; αCD40, αIgM (DMSO)
Flat; αCD40, αIgM (BAPTA-AM)
Nanopores (DMSO)
Nanopores (BAPTA-AM)
C
Thapsigargin
Live lymphocytes (%; Zombie NIR-)
Flat (DMSO)
Flat (Thapsigargin)
Flat; αCD40, αIgM (DMSO)
Flat; αCD40, αIgM (Thapsigargin)
Nanopores (DMSO)
Nanopores (Thapsigargin)
D
Cytochalasin D
Live lymphocytes (%; Zombie NIR-)
Flat (DMSO)
Flat (CytoD)
Flat; αCD40, αIgM (DMSO)
Flat; αCD40, αIgM (CytoD)
Nanopores (DMSO)
Nanopores (CytoD)
E
Jasplakinolide
Live lymphocytes (%; Zombie NIR-)
Flat (DMSO)
Flat (Jasplakinolide)
Flat; αCD40, αIgM (DMSO)
Flat; αCD40, αIgM (Jasplakinolide)
Nanopores (DMSO)
Nanopores (Jasplakinolide)
F
GsMTx-4
Live lymphocytes (%; Zombie NIR-)
Flat (DMSO)
Flat (GsMTx-4)
Flat; αCD40, αIgM (DMSO)
Flat; αCD40, αIgM (GsMTx-4)
Nanopores (DMSO)
Nanopores (GsMTx-4)
G
GsMTx4, Thapsigargin
Live lymphocytes (%; Zombie NIR-)
*
Flat (DMSO)
Flat (GsMTx-4, Thapsigargin)
Flat; αCD40, αIgM (DMSO)
Flat; αCD40, αIgM (GsMTx-4, Thapsigargin)
Nanopores (DMSO)
Nanopores (GsMTx-4, Thapsigargin)
H
GsMTx4, Cytochalasin D
Live lymphocytes (%; Zombie NIR-)
Flat (DMSO)
Flat (GsMTx-4, CytoD)
Flat; αCD40, αIgM (DMSO)
Flat; αCD40, αIgM (GsMTx-4, CytoD)
Nanopores (DMSO)
Nanopores (GsMTx-4, CytoD)
I
Ibrutinib
Live B cells (%; CD19+, Zombie NIR-)
J
BAPTA-AM
Live B cells (%; CD19+, Zombie NIR-)
***
K
Thapsigargin
Live B cells (%; CD19+, Zombie NIR-)
L
Cytochalasin D
Live B cells (%; CD19+, Zombie NIR-)
M
Jasplakinolide
Live B cells (%; CD19+, Zombie NIR-)
N
GsMTx-4
Live B cells (%; CD19+, Zombie NIR-)
O
GsMTx4, Thapsigargin
Live B cells (%; CD19+, Zombie NIR-)
P
GsMTx4, Cytochalasin D
Live B cells (%; CD19+, Zombie NIR-)
