# Supplementary material for: B Cells are Activated via a Nanoporous Interface that Stabilizes Microvilli and Engages Mechanosensitive Ion Channels
Source: Adv Sci (Weinh). 2026 Aug 3:e76869. Online ahead of print. doi: 10.1002/advs.76869 (PMC13430938; doi:10.1002/advs.76869)
Supplement: Supplementary file 1 — Supporting File 1: advs76869‐sup‐0001‐SuppMat.docx. [file ADVS-9999-e76869-s002.docx]

Supplementary Materials for

**B cells are activated via a nanoporous interface that stabilizes microvilli and engages mechanosensitive ion channels**

Nozie D. Aghaizu* *et al.*

*Corresponding author. [dominic.aghaizu@hu-berlin.de](mailto:dominic.aghaizu@hu-berlin.de)

**This PDF file includes:**

Figures S1 to S6

Table S1 (reagents and resources)


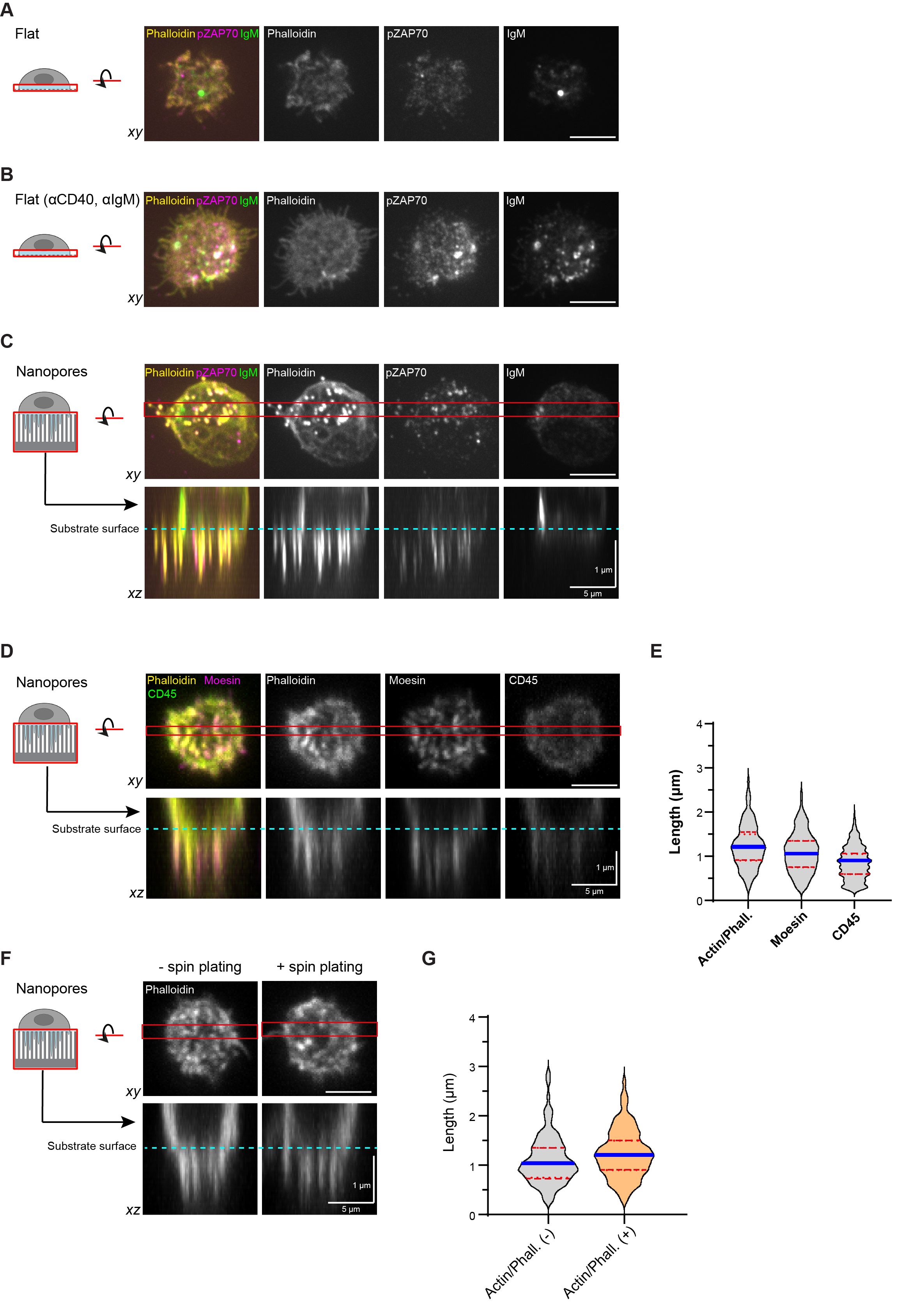


Figure S1.

**B cells extend actin-filled microvilli into nanoporous substrates.** B cell morphology based on phalloidin staining (yellow) with additional pZAP70 (magenta) and IgM immunolabelling (green) 30 mins after seeding on flat (**A**), flat (αCD40, αIgM) (**B**), and nanoporous substrates (**C**). (**D**) B cell morphology based on phalloidin staining (yellow) with additional moesin (magenta) and CD45 immunolabelling (green) 30 mins after seeding on nanoporous substrates. (**E**) Length of phalloidin, moesin, and CD45 signals within phalloidin^+ve^ microvilli. (**F**) B cell morphology based on phalloidin staining (grayscale) 30 mins after seeding on nanoporous substrates with or without prior spin plating step at 50 *g* for 5 min. (**G**) Length of B cell microvilli protrusions into nanoporous substrates based on phalloidin staining with (+) or without (-) prior spin plating step. Scale bars, 5 µm (A-D, F) unless stated otherwise. Violin plots show mean (blue horizontal bar) ± SD (red dotted horizontal bar) (E, G). *N* = 1 biological repeat (independent blood donor), *n* = total 55 cells imaged for microvilli analysis on nanoporous substrate (E, G).


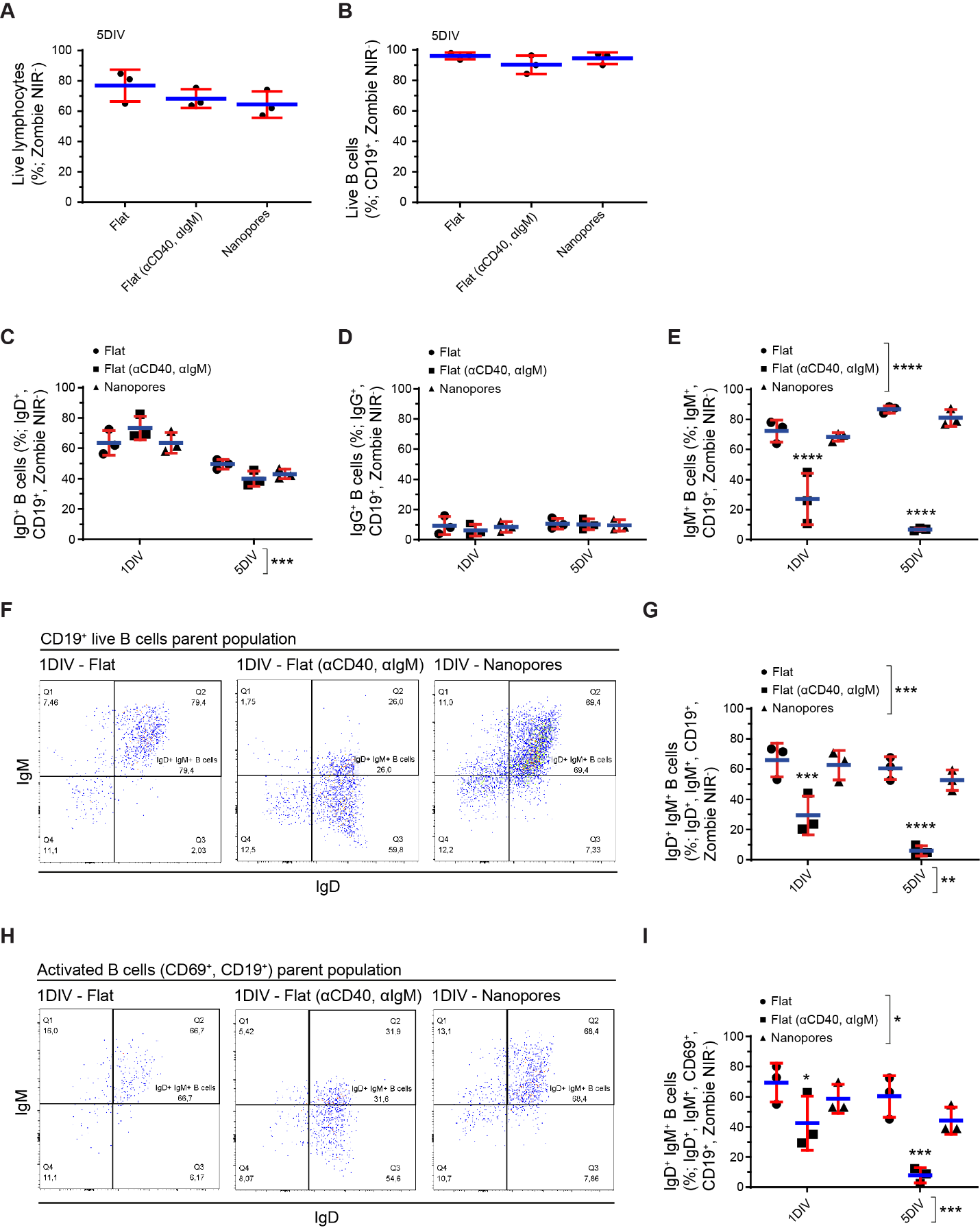


Figure S2.

**BCR phenotyping and class-switch analysis by flow cytometry.** Cell surface expression of IgD, IgG, and IgM BCR variants on B cells exposed to flat, flat (αCD40, αIgM), and nanoporous substrate. (**A**) Viability analysis of gated lymphocytes stated as % ZombieNIR^-ve^ of parent single lymphocyte population. (**B**) Purity assessment of B cells (CD19^+ve^) as opposed to T cells (CD3^+ve^) within cell cultures stated as % of parent live lymphocyte population. (**C**) IgD cell surface expression on B cells (IgD^+ve^, CD19^+ve^) stated as % of parent live B cell population following one and five days of *in vitro* incubation. (**D**) IgG cell surface expression on B cells (IgG^+ve^, CD19^+ve^) stated as % of parent live B cell population following one and five days of *in vitro* incubation. (**E**) IgM cell surface expression on B cells (IgM^+ve^, CD19^+ve^) stated as % of parent live B cell population following one and five days of *in vitro* incubation. (**F**) Representative IgM vs. IgD expression scatter plots for B cells exposed to flat, flat (αCD40, αIgM), and nanoporous substrate for 1 DIV with CD19^+ve^ live B cells used as parent population. (**G** IgD^+ve^ IgM^+ve^ double positive B cells within CD19^+ve^ live B cell parent population on different substrates compared at 1 and 5 DIV. (**H**) Representative IgM vs. IgD expression scatter plots for B cells exposed to flat, flat (αCD40, αIgM), and nanoporous substrate for 1 DIV with activated CD69^+ve^ CD19^+ve^ live B cells used as parent population. (**I**) IgD^+ve^ IgM^+ve^ double positive B cells within activated CD69^+ve^ CD19^+ve^ live B cell parent population on different substrates compared at 1 and 5 DIV. *N* = 3 biological repeats (independent blood donors). One-way ANOVA (A, B); ***p < 0.001. Two-way ANOVA (C-E, G, I); *p < 0.05; **p < 0.01; ***p < 0.001; ****p < 0.0001.


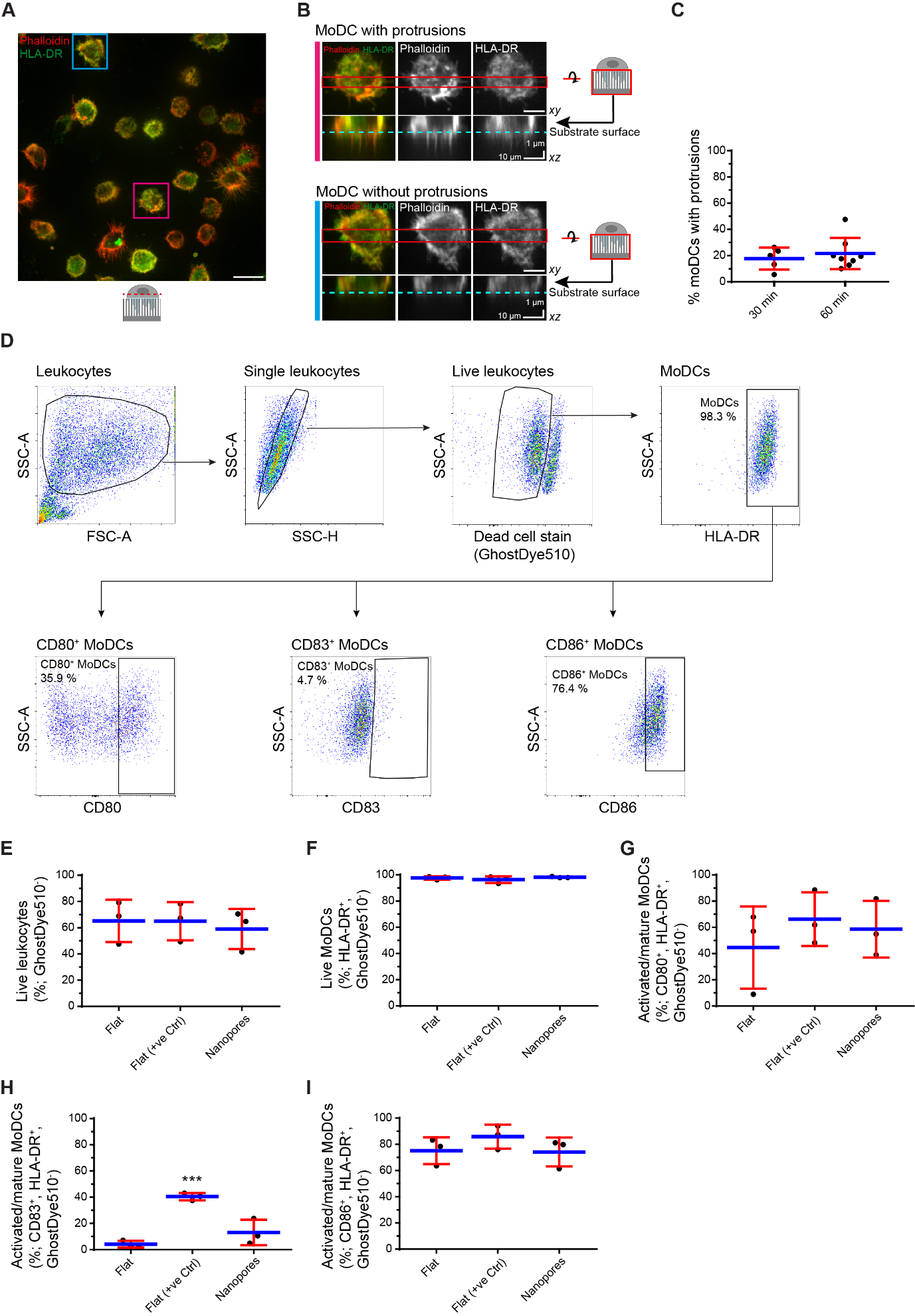


Figure S3.

**Assessing dendritic cell behaviour on nanoporous substrates.** Monocyte-derived dendritic cells (moDCs) generated from frozen PBMCs were exposed to nanoporous substrates and responses were assessed by fluorescence microscopy and flow cytometry. (**A**) moDC morphology at low magnification at two different *z* depths above and below the level of the nanoporous surface based on phalloidin staining (red) and HLA-DR immunostaining (green). ROIs (blue, magenta) are enlarged in (B). (**B**) moDC morphology at high magnification based on phalloidin staining (red) and HLA-DR immunostaining (green). Example moDCs with or without protrusions into nanoporous substrate were derived from ROIs (magenta and blue respectively) in (A). (**C**) % of moDCs exhibiting protrusions into nanopores following 30 min (*n* = 84 total cells) and 60 min incubation (*n* = 245 total cells) on nanoporous substrate. (**D**) Representative flow cytometry gating strategy for moDC cell surface marker analysis at 24 hrs on nanoporous substrate. (**E**) Viability analysis of gated leukocytes stated as % GhostDye510^-ve^ of parent single leukocyte population. (**F**) Purity assessment of moDCs (HLA-DR^+ve^) within cell cultures stated as % of parent live leukocyte population (see also D). (**G**) Cell surface expression of the activation/maturation marker CD80 on moDCs (CD80^+ve^, HLA-DR^+ve^) stated as % of parent live moDC population following one day of *in vitro* incubation. (**H**) Cell surface expression of the activation/maturation marker CD83 on moDCs (CD83^+ve^, HLA-DR^+ve^) stated as % of parent live moDC population following one day of *in vitro* incubation. (**I**) Cell surface expression of the activation/maturation marker CD86 on moDCs (CD86^+ve^, HLA-DR^+ve^) stated as % of parent live moDC population following one day of *in vitro* incubation. *N* = 3 biological repeats (independent blood donors). One-way ANOVA (E-I); ***p < 0.001.

**
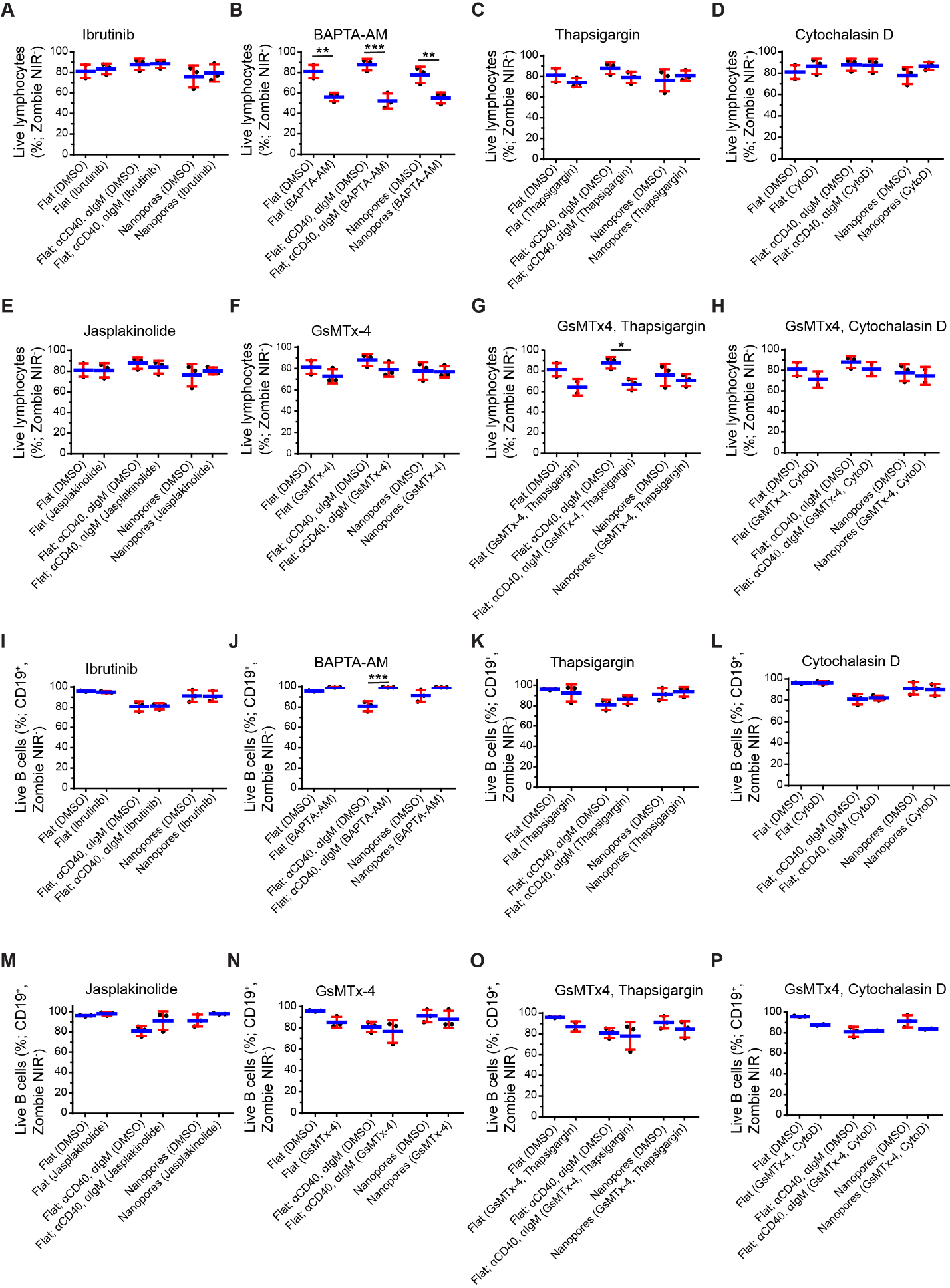
**

**Figure S4.**

**B cell culture viability and purity following pharmacological screening.** (**B**) Viability analysis of gated lymphocytes stated as % ZombieNIR^-ve^ of parent single lymphocyte population in presence of Ibrutinib (**A**), BAPTA-AM (**B**), Thapsigargin (**C**), Cytochalasin D (**D**), Jasplakinolide (**E**), GsMTx-4 (**F**), GsMTX-4/Thapsigargin (**G**), and GsMTx-4/Cytochalasin D (**H**). Purity assessment of B cells (CD19^+ve^) as opposed to T cells (CD3^+ve^) within cell cultures stated as % of parent live lymphocyte population in presence of Ibrutinib (**I**), BAPTA-AM (**J**), Thapsigargin (**K**), Cytochalasin D (**L**), Jasplakinolide (**M**), GsMTx-4 (**N**), GsMTX-4/Thapsigargin (**O**), and GsMTx-4/Cytochalasin D (**P**). *N* = 3 biological repeats (independent blood donors). One-way ANOVA (A-P); *p < 0.05; **p < 0.01; ***p < 0.001.


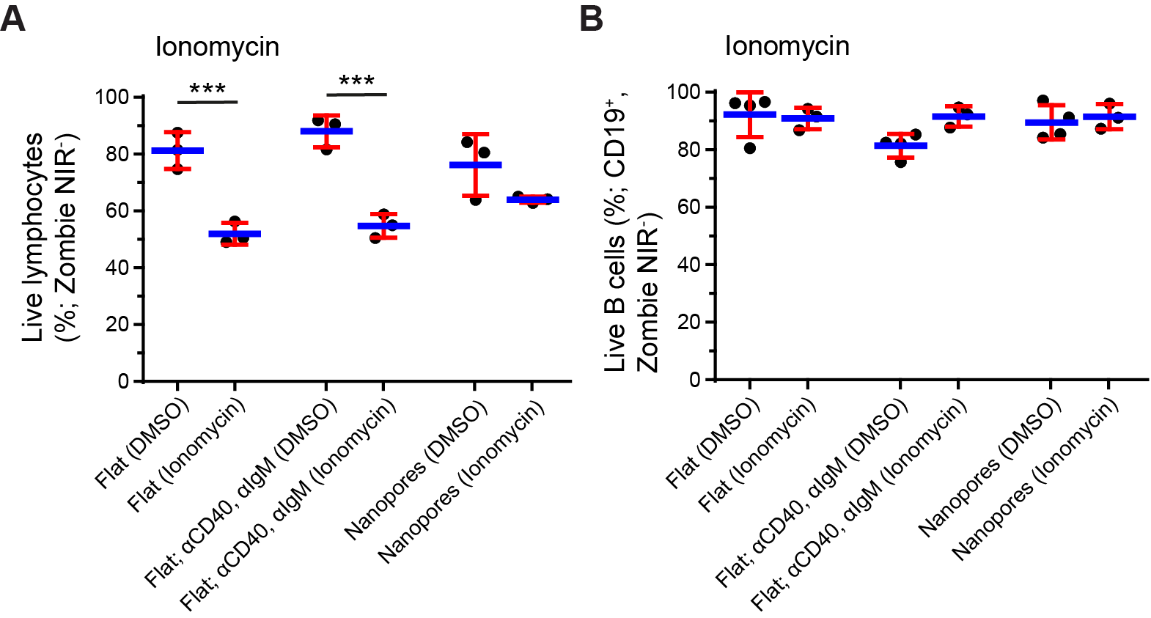


**Figure S5.**

**B cell flow cytometry quality control metrics following ionomycin treatment.** B cells were incubated with 1 µM ionomycin or DMSO vehicle control for 24 hrs on flat, flat (αCD40, αIgM), and nanopore substrates and subsequently harvested and processed for analysis by flow cytometry. (**A**) Viability analysis of gated lymphocytes stated as % ZombieNIR^-ve^ of parent single lymphocyte population in presence of ionomycin or DMSO vehicle control. (**B**) Purity assessment of B cells (CD19^+ve^) as opposed to T cells (CD3^+ve^) within cell cultures stated as % of parent live lymphocyte population in presence of ionomycin or DMSO vehicle control. One-way ANOVA (A, B); ***p < 0.001.

**
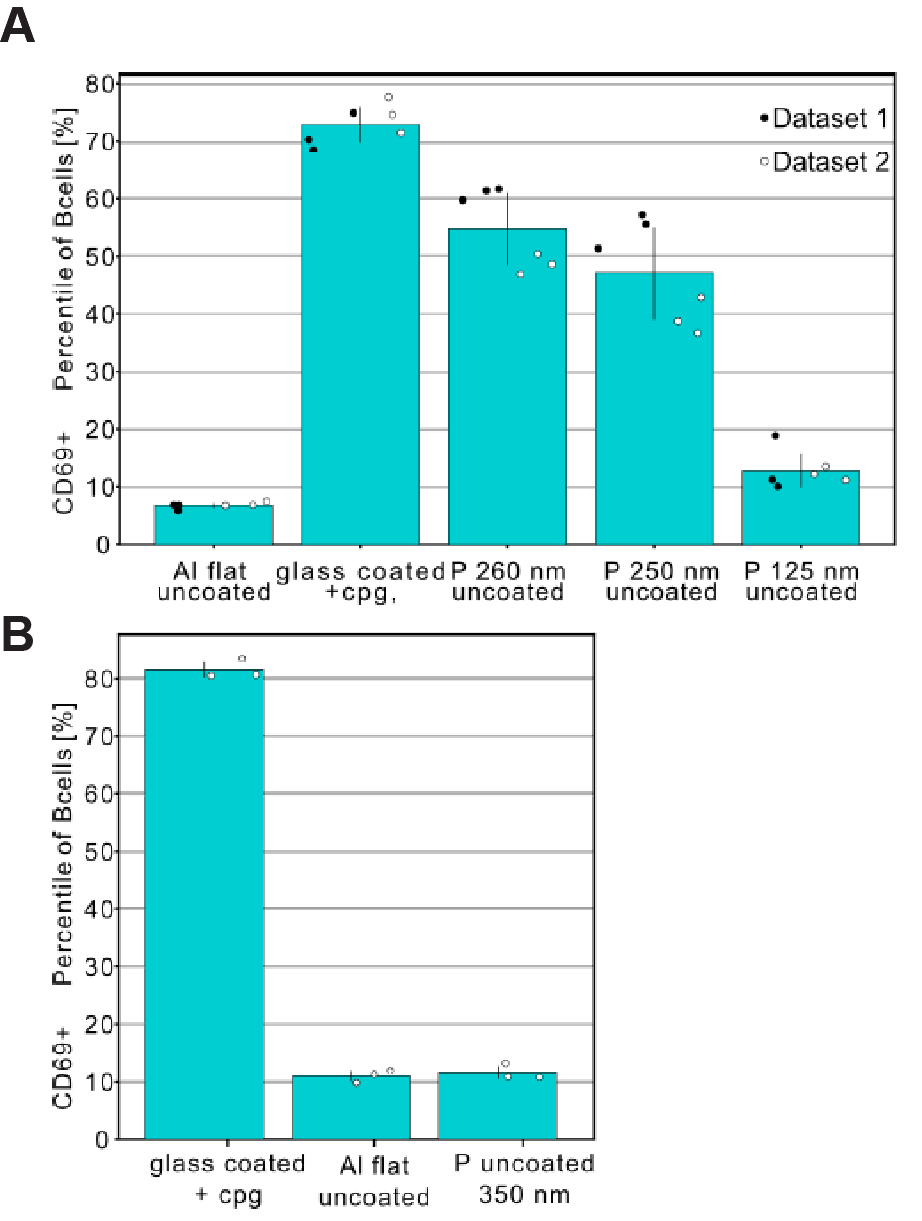
**

**Figure S6.**

**Nanopore-mediated B cell activation is pore size dependent.** (**A**) Percentage of human primary B cells expressing the activation marker CD69 following incubation for 24 hours on different substrates as assessed by flow cytometry. (**B**) Percentage of human primary B cells expressing the activation marker CD69 following incubation for 24 hours on different substrates as assessed by flow cytometry. “Al(uminium) flat uncoated” refers to flat negative control surface; “glass coated + cpg” refers to a positive control glass surface coated with αIgM and αCD40 antibodies and in additional presence of the adjuvant CpG; “P” refers to nanoporous substrate with pore diameters as indicated.

**Reagents and resources**

| **Reagent/resource** | **Source** | **Identifier** |
| --- | --- | --- |
| Antibodies |  |  |
| beta actin | Synaptic Systems | 251006, RRID:AB_2782985 |
| CD45 | Abcam | ab30470, RRID:AB_726544 |
| goat anti human IgM Alexa Fluor 488 | Jackson ImmunoResearch | 109-547-043, RRID: AB_2337855 |
| goat anti mouse IgG Dylight 405 | ThermoFisher | 35500BID, RRID:AB_2533208 |
| goat anti rabbit IgG Alexa Fluor 568 | ThermoFisher | A-11036, RRID: AB_10563566 |
| human CD19-AF647 | Biolegend | 302220, RRID:AB_389335 |
| human CD19-BV421 | Biolegend | 363018, RRID:AB_2564227 |
| human CD3-PE | Biolegend | 317308, RRID:AB_571913 |
| human CD69-APC | Biolegend | 310910, RRID:AB_314845 |
| human CD80-BV421 | Biolegend | 305222, RRID:AB_2564407 |
| human CD80-PE/Dazzle594 | Biolegend | 305230, RRID:AB_2566489 |
| human CD83-APC/Vio770 | Miltenyi | 130-110-506, RRID: AB_2659318 |
| human CD83-FITC | Biolegend | 305306, RRID:AB_314514 |
| human CD86-BV650 | Biolegend | 305428, RRID:AB_2563823 |
| human HLA-DR-BV711 | Biolegend | 307644, RRID:AB_2562913 |
| human HLA-DR PE | Miltenyi | 130-111-942, RRID:AB_2726058 |
| human IgD-BV605 | Biolegend | 348232, RRID:AB_2563337 |
| human IgG-PE/Cy7 | Biolegend | 410722, RRID:AB_2750227 |
| human IgM-BV421 | Biolegend | 314516, RRID:AB_2561443 |
| moesin | Abcam | ab52490, RRID:AB_881245 |
| mouse anti human CD40 | Biolegend | 334343, RRID:AB_2566580 |
| mouse anti human IgM | Southern Biotech | 9022-08, RRID:AB_2796585 |
| pBTK | Fisher Scientific | NBP1-78295, RRID: AB_11032627 |
| pZAP70 | Cell Signaling | 2717S, RRID:AB_2218658 |
|  |  |  |
| Chemicals, solutions, media |  |  |
| Tween-20 | BioRad | 1706531 |
| MES | Sigma | M3671 |
| PBS (without Ca^2+^, Mg^2+^) | VWR | 392-0434 |
| PENICILLIN -STREPTOMYCIN | VWR | SIALP4333-100ML |
| EDTA | VWR | MDTC46-034-CI |
| RPMI 1640 | PAN Biotech | P04-17500 |
| RPMI 1640 w/o (Phenol red) | PAN Biotech | P04-16516 |
| FBS | ATCC-LGC Standards | ATCC-30-2020 |
| HEPES | ThermoFisher | 15630080 |
| L-Glutamin | ThermoFisher | 25030081 |
| DMSO | Sigma | D8418-50ML |
| BSA | Sigma | A8806-1G |
| X-Vivo 15 | Lonza | 02-060Q |
| PFA | Electron Microscopy Sciences | 15714 |
| NaCl | Sigma | S9888-25G |
| Glucose | Sigma | G8270-100G |
| EGTA | Sigma | E3889-10G |
| MgCl_2_ | Sigma | 63069-100ML |
| NH_4_Cl | Sigma | 213330-25G |
| Normal goat serum | Biolegend | 927502 |
| Triton x-100 | Sigma | X100-100ML |
| Prolong gold antifade mountant | Molecular Probes | 11559306 |
| HBSS | PAN Biotech | P04-33500 |
| FC block | Miltenyi | 130-059-901 |
|  |  |  |
| Critical commercial reagents, kits, assays |  |  |
| B cell isolation kit II | Miltenyi Biotec | 130-091-151 |
| CD14^+^ microbeads | Miltenyi Biotec | 130-050-201 |
| LS separation columns | Miltenyi | 130-042-401 |
| Lymphoprep density gradient medium | Stemcell Technologies | 18060 |
| Midi MACS separator | Miltenyi | 130-042-301 |
| SepMate tube, 50 ml | Stemcell Technologies | 85460 |
|  |  |  |
| Cytokines |  |  |
| GM‑CSF | PeproTech | AF-300-03 |
| IL-1β | PeproTech | AF-200-01B |
| IL-4 | PeproTech | AF-200-04 |
| IL-6 | Miltenyi Biotech | 130-093-929 |
| Prostaglandin E2 (PGE2) | Enzo | BML-PG007-001 |
| TNF-α | PeproTech | AF-300-01A |
|  |  |  |
| Equipment |  |  |
| FACS Aria II | BD | / |
| ID7000 flow cytometer | Sony | / |
| Leitz Laborlux S microscope | Leitz | / |
| Plasma cleaner Zepto model 2 | Diener | / |
| Spinning disc microscope, FV1000 | Visitron/Olympus | / |
|  |  |  |
| Experimental models |  |  |
| Peripheral blood / buffy coat (for PBMCs, B cells, monocytes) | DRK-Blutspendedienst Nord-Ost gemeinnützige GmbH | NA |
|  |  |  |
| Materials |  |  |
| Nanoporous aluminium sheets (FlexiPor, pore depth ~5-10 µm, pore diameter ~260 nm) | Smartmembranes | FlexiPorS0.26-(5-10) (145-2023_08_15) |
| Flat aluminium sheets, AL 99,98%, Toyo Aluminium 99,98% gereinigt | Smartmembranes | Referenzprobe Aluminiumblech 99.98%, 20 x 30 cm, 200 µm |
|  |  |  |
| Other fluorescent reagents |  |  |
| Fluo4-AM | Invitrogen | F14201 |
| Ghost Dye Violet 510 | Cell Signalling | 59863S |
| Phalloidin Alexa Fluor 647 | ThermoFisher | A22287 |
| Zombie NIR | Biolegend | 423106 |
|  |  |  |
| Pharmacological reagents |  |  |
| BAPTA-AM | Tocris | 2787 |
| Cytochalasin D | ThermoFisher | PHZ1063 |
| GsMTx-4 | Hölzel Biotech | HY-P1410-5mg |
| Ibrutinib | Fisher Scientific | 16474338 |
| Ionomycin | Sigma | I3909 |
| Jasplakinolide | Sigma | 420127-50UG |
| Thapsigargin | Fisher Scientific | T7458 |
|  |  |  |
| Software and algorithms |  |  |
| Ca^2+^ time lapse live imaging analysis | This paper (W. Weber) | Available on request |
| Fiji/ImageJ | NIH | <https://imagej.nih.gov/ij/> |
| Flowjo v10.10.0 | Treestar | <https://www.flowjo.com/> |
| GraphPad Prism | GraphPad | <https://www.graphpad.com/scientificsoftware/prism/> |
| Illustrator | Adobe | <https://www.adobe.com/Illustrator> |
| Microvilli quantification | This paper (A. Polat, W. Weber, N.D. Aghaizu) | Available on request |
| Trackmate | ImageJ plugin | <https://imagej.net/plugins/trackmate/> |
| Visiview Software | Visitron Systems | <https://www.visitron.de/products/visiviewr-software> |
| Spyder v5 | Spyder | <https://www.spyder-ide.org/> |
