# Supplementary figures and images for: B Cells are Activated via a Nanoporous Interface that Stabilizes Microvilli and Engages Mechanosensitive Ion Channels
Source: Adv Sci (Weinh). 2026 Aug 3:e76869. Online ahead of print. doi: 10.1002/advs.76869 (PMC13430938; doi:10.1002/advs.76869)

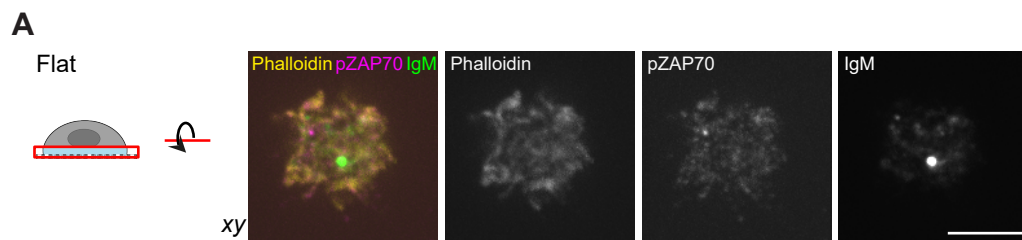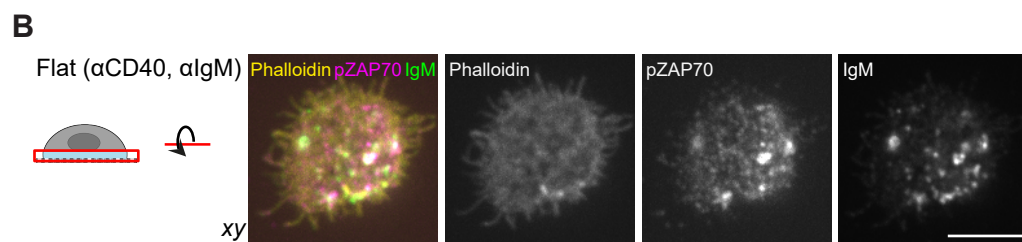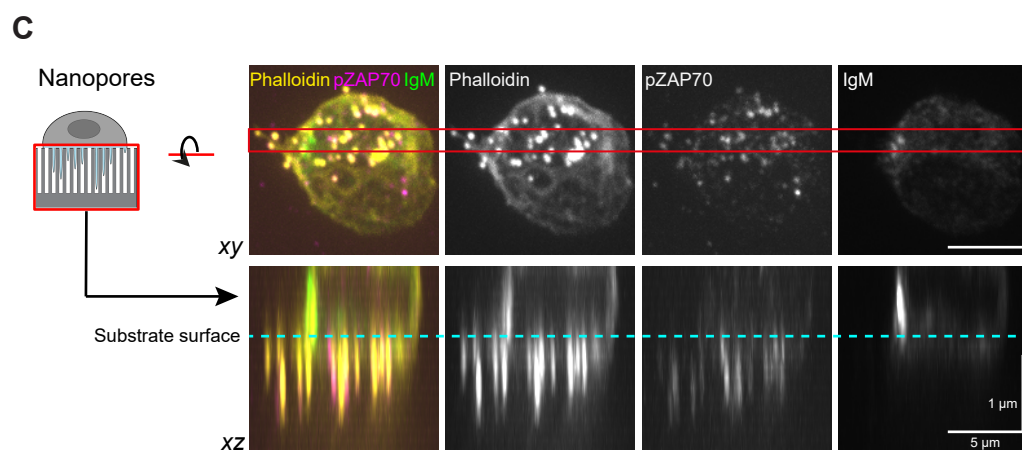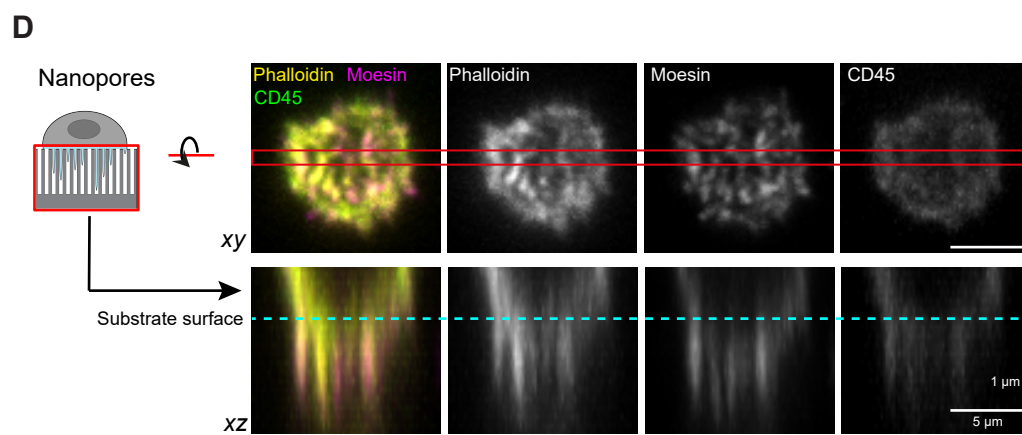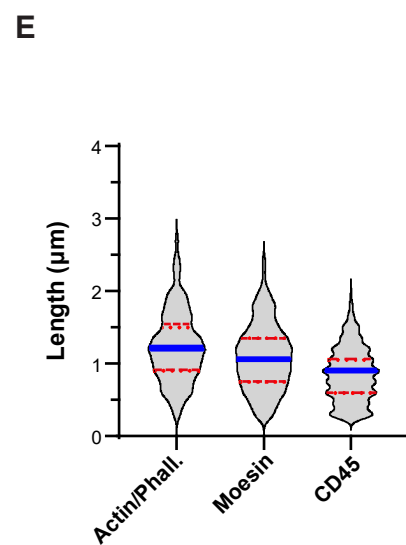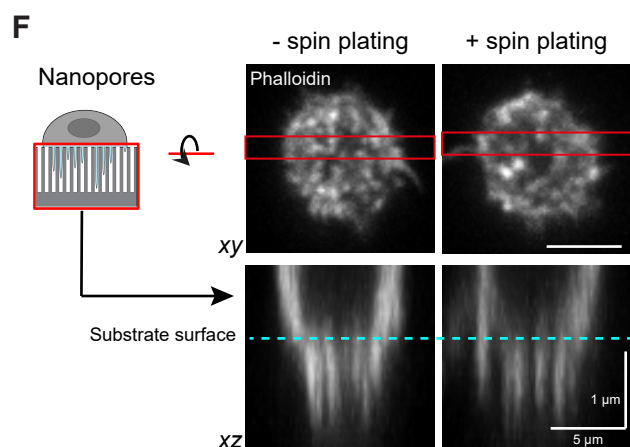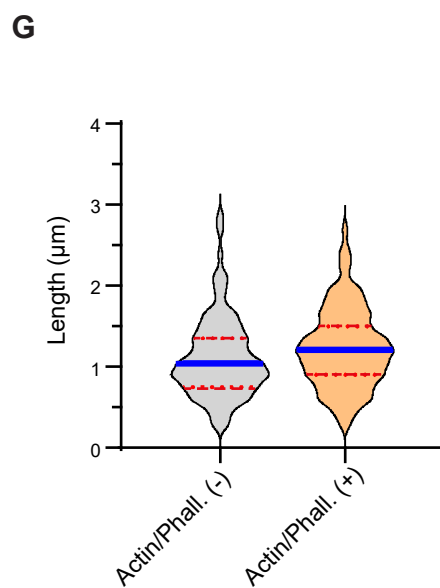

Supplement: Supplementary file 2 — Supporting File 2: advs76869‐sup‐0002‐FigureS1_1.pdf. [file ADVS-9999-e76869-s005.pdf]

**A**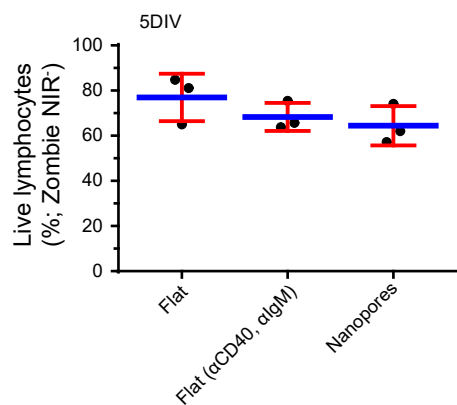**B**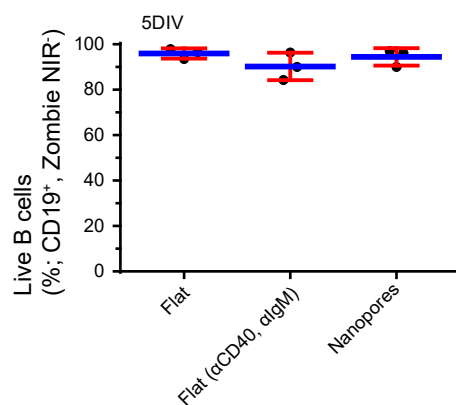**C**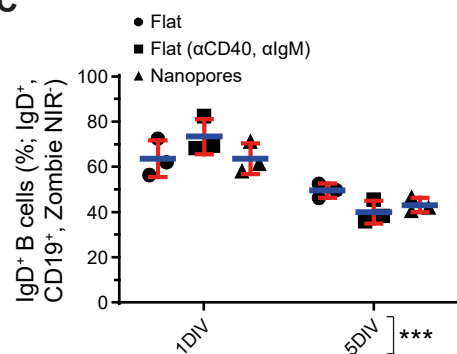**D**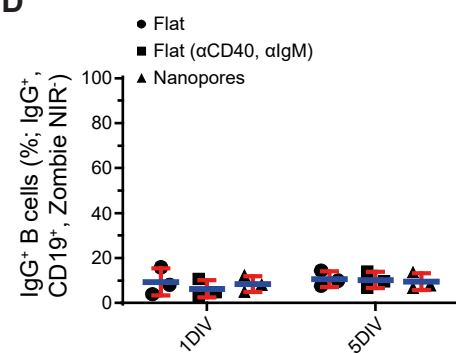**E**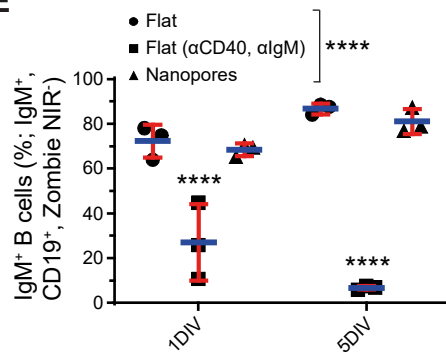**F**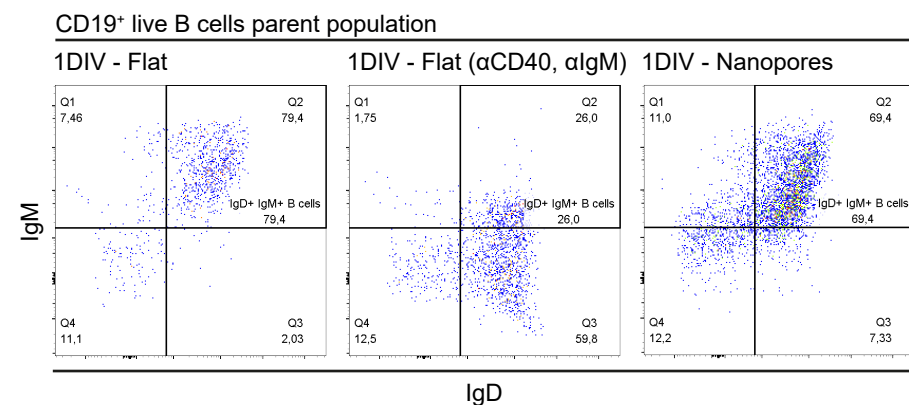**G**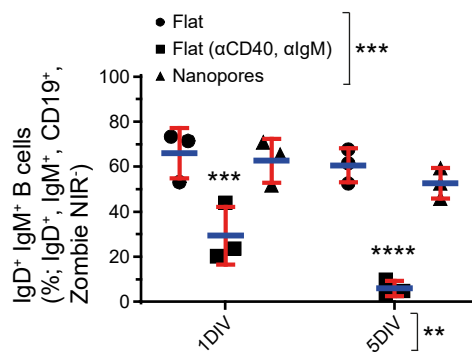**H**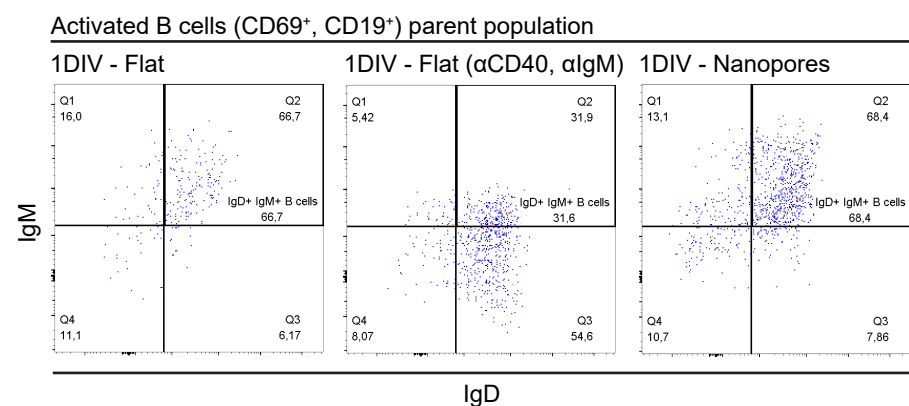**I**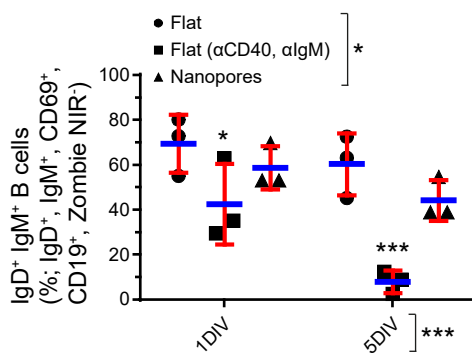

Supplement: Supplementary file 3 — Supporting File 3: advs76869‐sup‐0003‐FigureS2_1.pdf. [file ADVS-9999-e76869-s004.pdf]

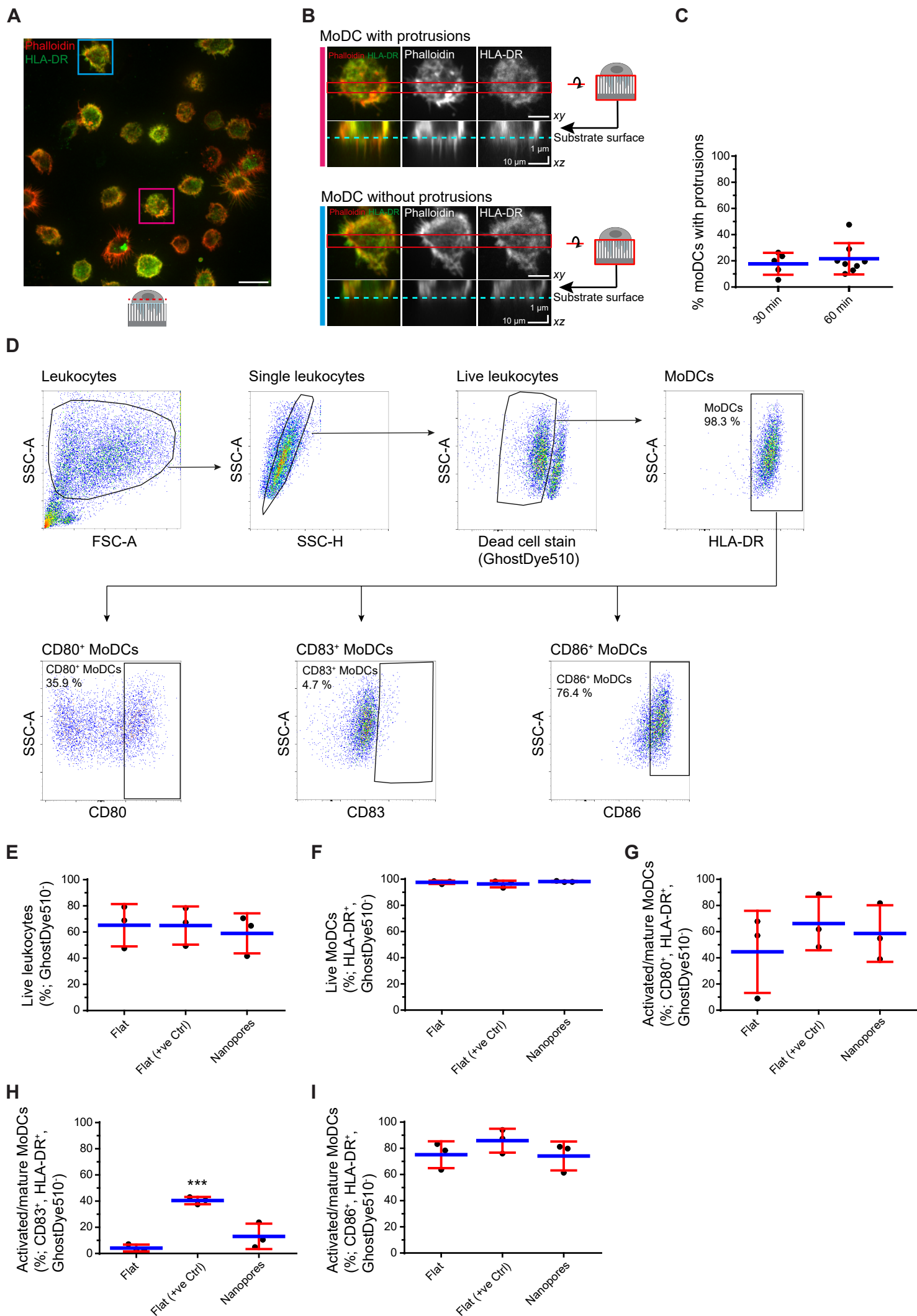

Supplement: Supplementary file 4 — Supporting File 4: advs76869‐sup‐0004‐FigureS3_1.pdf. [file ADVS-9999-e76869-s007.pdf]

**A**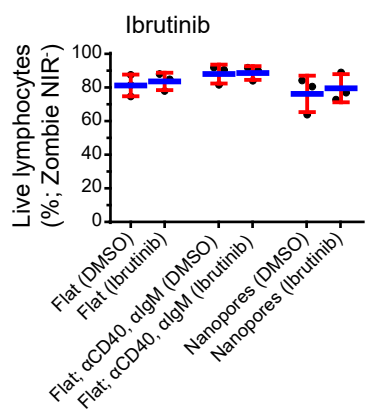**B**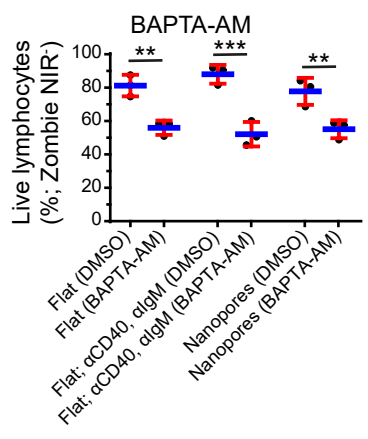**C**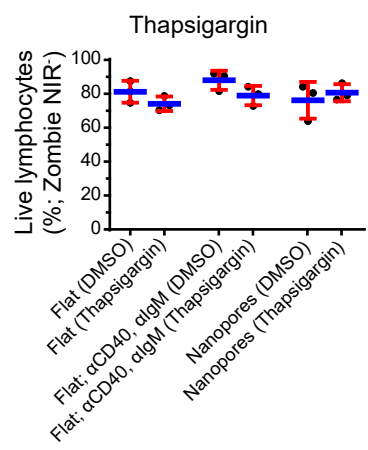**D**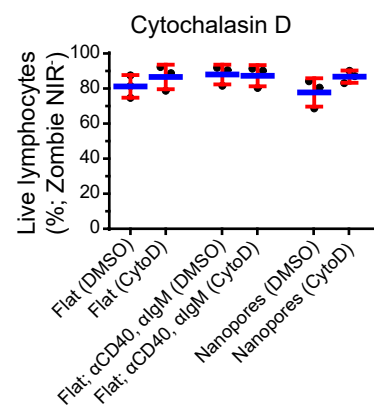**E**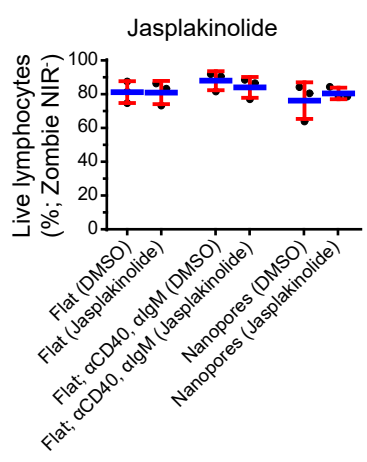**F**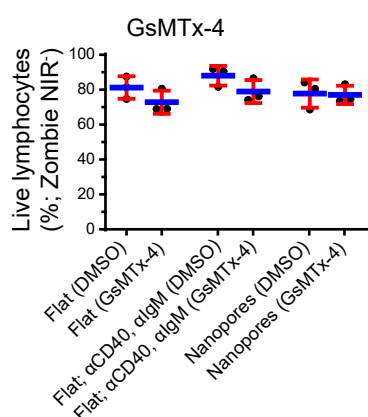**G**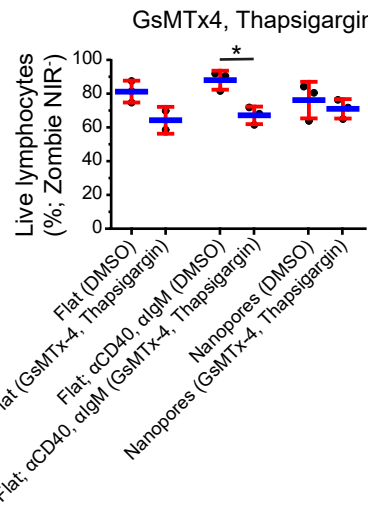**H**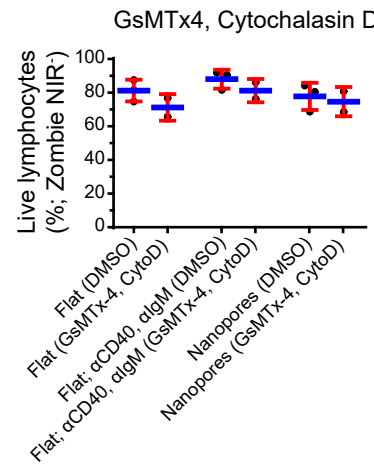**I**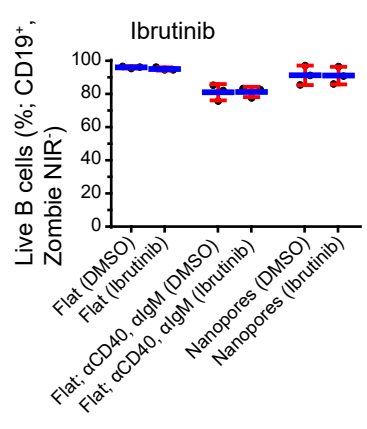**J**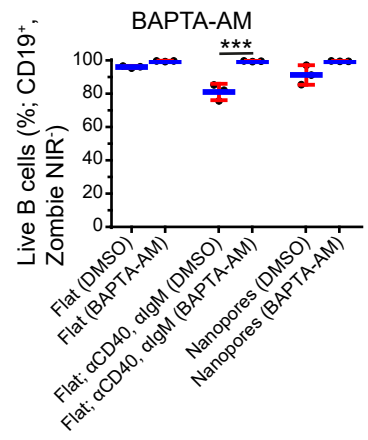**K**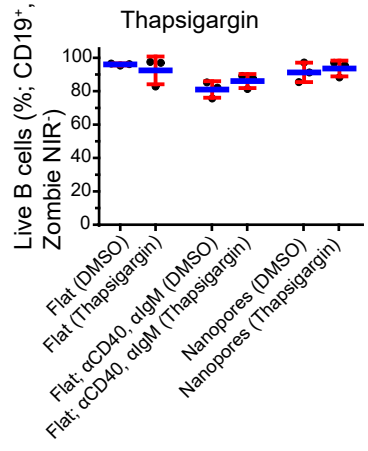**L**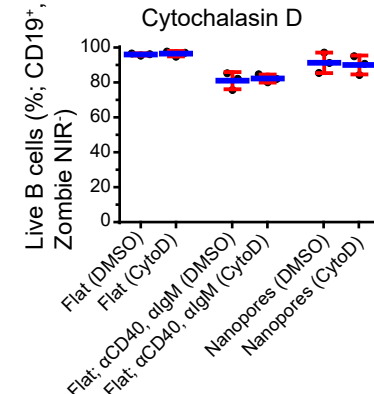**M**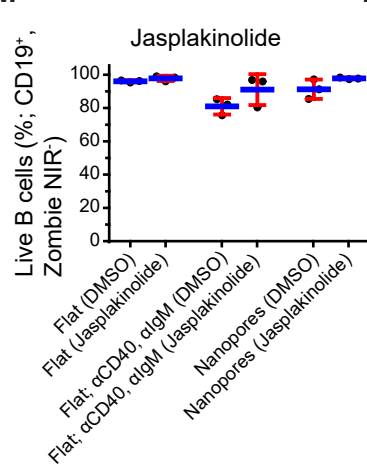**N**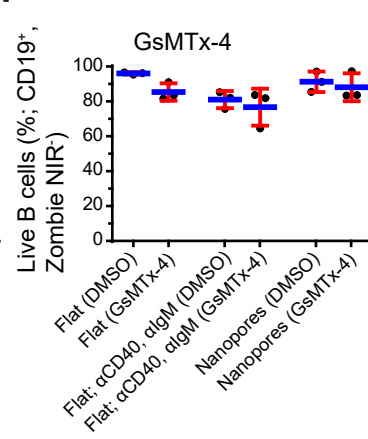**O**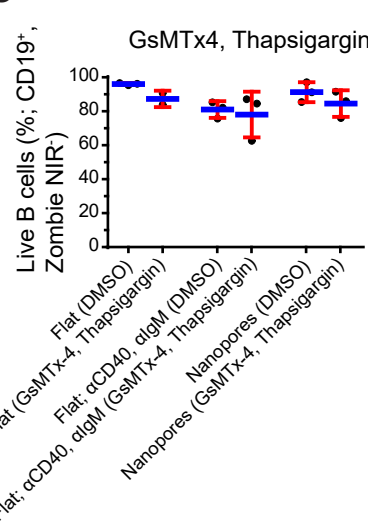**P**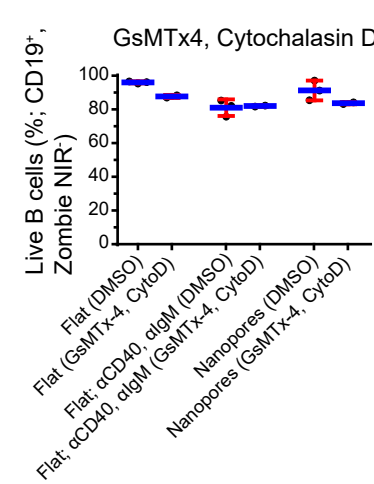

Supplement: Supplementary file 5 — Supporting File 5: advs76869‐sup‐0005‐FigureS4.pdf. [file ADVS-9999-e76869-s001.pdf]

**A**

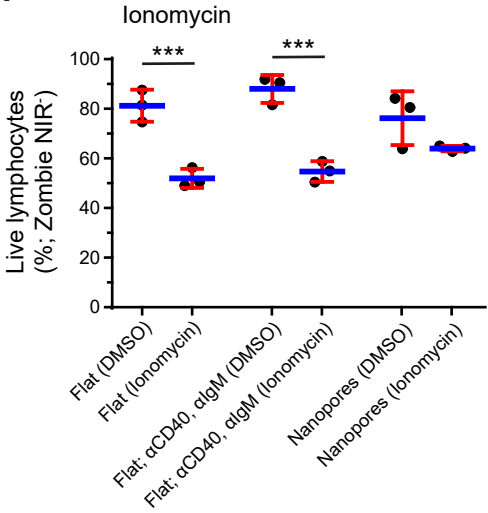

**B**

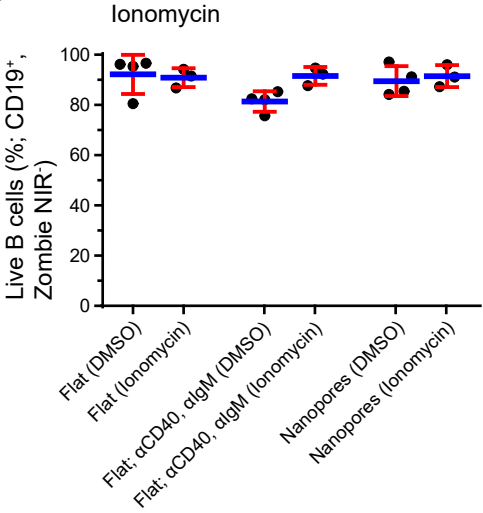

Supplement: Supplementary file 6 — Supporting File 6: advs76869‐sup‐0006‐FigureS5.pdf. [file ADVS-9999-e76869-s003.pdf]

A

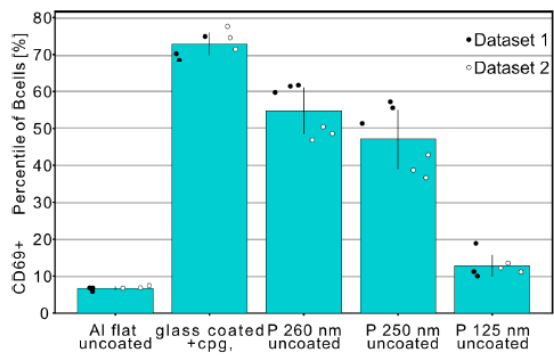

B

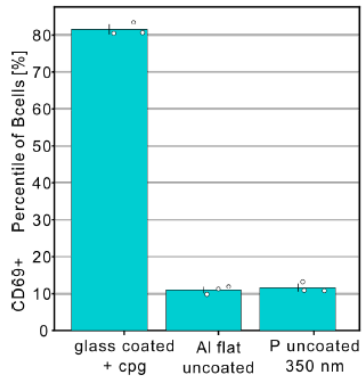

Supplement: Supplementary file 7 — Supporting File 7: advs76869‐sup‐0007‐FigureS6.pdf. [file ADVS-9999-e76869-s006.pdf]
